# Text Messaging and Video Stories to Support Hypertension Self-Management in Black Veterans: A Randomized Clinical Trial

**DOI:** 10.1001/jamanetworkopen.2025.41342

**Published:** 2025-11-05

**Authors:** Sarah L. Cutrona, Sarah E. McDannold, Kathryn L. DeLaughter, Gemmae M. Fix, Stephanie L. Shimada, Barbara G. Bokhour, Howard S. Gordon, Charlene Pope, Bridget M. Smith, Ndindam Ndiwane, Jessica Gardner, Diana C. DeFelice, Ece S. Bal, Judith A. Long

**Affiliations:** 1 Center for Health Optimization and Implementation Research, Veterans Affairs (VA) Bedford Healthcare System, Bedford, Massachusetts; 2 Department of Population and Quantitative Health Sciences, University of Massachusetts Chan Medical School, Worcester; 3 Boston University Chobanian and Avedisian School of Medicine, Boston, Massachusetts; 4 Boston University School of Public Health, Boston, Massachusetts; 5 Jesse Brown VA Medical Center, Center of Innovation for Complex Chronic Healthcare, Chicago, Illinois; 6 Division of Academic Internal Medicine and Geriatrics, Department of Medicine, University of Illinois College of Medicine, Chicago; 7 Ralph J. Johnson VA Health Care System, Charleston, South Carolina; 8 College of Nursing, Medical University of South Carolina (MUSC), Charleston; 9 Spinal Cord Injuries and Disorders National Program Office, US Department of Veterans Affairs, Washington, DC; 10 Corporal Michael J. Crescenz VA Center for Healthcare Evaluation, Research, and Promotion, Philadelphia, Pennsylvania; 11 Division of General Internal Medicine, Perelman School of Medicine, University of Pennsylvania, Philadelphia; 12 Leonard Davis Institute of Health Economics, University of Pennsylvania, Philadelphia

## Abstract

**Importance:**

Hypertension treatment advances have resulted in improvements in blood pressure control and self-management. Yet, disparities persist for Black veterans.

**Objective:**

To examine the impact of a narrative-informed texting intervention with educational content and bidirectional text messaging on blood pressure and hypertension self-management.

**Design, Setting, and Participants:**

This nonblinded, 2-arm randomized clinical trial recruited participants from March 2021 to July 2022 at 2 Veterans Affairs (VA) medical centers (1 in Chicago, Illinois, and 1 in Philadelphia, Pennsylvania); follow-up was completed in May 2023. Participants were Black veterans with at least 1 medication for hypertension, with oversampling of female veterans. Participants were randomly assigned to the intervention arm or control arm. Data analysis was based on the intention-to-treat principle.

**Intervention:**

Those assigned to the intervention group received the Continuing the Conversation intervention, which involved watching 5 videos (5-7 minutes each) of Black veterans sharing stories of their challenges and successes with managing hypertension. Participants selected a favorite storyteller and were enrolled in a 6-month texting protocol wherein they received 3 types of text messages: narrative (quotations from storytellers), educational, and bidirectional. The comparison or control arm received 6 months of bidirectional text messages (BTMs) only.

**Main Outcomes and Measures:**

Change in systolic and diastolic blood pressure from baseline to 6-month follow-up was the primary outcome. Secondary outcomes included change in self-reported hypertension management activities (medication adherence, diet, physical activity). All outcomes were prespecified. Difference-in-differences (DID) regression models assessed whether the intervention arm had a 6-month improvement larger than that in the control arm. Engagement (6-month response rates to BTMs) and motivation of participants were also evaluated.

**Results:**

Of the 600 Black veterans enrolled (469 males [78.7%]; mean [SD] age, 64.0 [9.1] years), 516 veterans (86.0%; 262 in intervention arm, 254 in control arm) completed the 6-month follow-up. There was no significant difference between arms in mean change of systolic (DID, −0.8 mm Hg; 95% CI, –3.9 to 2.3 mm Hg; *P* = .62) or diastolic (DID, 0.4 mm Hg; 95% CI, −1.6 to 2.4 mm Hg; *P* = .70) blood pressure or in secondary outcomes. Mean systolic (−2.1 mm Hg; 95% CI, –3.7 to −0.6 mm Hg; *P* = .006) and diastolic (−1.8 mm Hg; 95% CI, –2.8 to −0.8 mm Hg; *P* = .001) blood pressure measurements decreased significantly for the entire cohort. In the intervention arm, 221 of 239 participants (92.5%) agreed with the statement: “I could identify with the Veteran in the video.” Across both arms, the response rate to BTMs was 57.5% (11 427 of 19 875 possible opportunities to respond), and 60.7% (10 564 of 17 407 possible opportunities to respond) among those completing follow-up. Of 516 veterans, 410 (79.5%) described text messages as helpful motivators for improving health.

**Conclusions and Relevance:**

In this randomized clinical trial of video stories followed by narrative, educational, and BTMs to support hypertension self-management, this intervention did not improve blood pressure compared with receiving BTMs alone. Text messages supported engagement and enhanced motivation for Black veterans.

**Trial Registration:**

ClinicalTrials.gov Identifier: NCT03970590

## Key Points

**Question:**

Can a narrative-informed texting intervention improve blood pressure and hypertension self-management for Black veterans?

**Findings:**

In a randomized clinical trial including 600 Black veterans with hypertension, there was no significant difference in systolic and diastolic blood pressure change between the intervention (ie, video stories plus narrative, educational, and bidirectional text messages) and control (ie, bidirectional text messages alone) groups. Veterans described text messages as helpful motivators for improving health.

**Meaning:**

In this trial, an enhanced texting intervention with video storytellers and narrative messages did not improve blood pressure compared with bidirectional text messaging alone, but it supported engagement and motivation for Black veterans.

## Teaser

This randomized clinical trial examines the effect of a video-based storytelling and text messaging intervention on blood pressure self-management among Black veterans with hypertension.

## Introduction

Hypertension affects more than half of Black adults in the US, with disparities in diagnosis, management, and outcomes.^1,2,3^ There is a higher prevalence of diagnosed hypertension in Black adults (34.4%) compared with White (27.4%) or Asian adults (14.5%)^4^; factoring in undiagnosed hypertension brings total prevalence estimates to more than 55% for Black adults.^1^ Hypertension in Black Americans develops earlier in life, is more severe, and yields disproportionately higher rates of adverse outcomes.^5,6^ Inadequate hypertension control contributes to higher risk of fatal stroke, end-stage kidney disease, and mortality from cardiovascular disease.^6^ For Black veterans, military experience and exposures can impact development of hypertension and experiences with self-management.^7^

Adoption of a heart-healthy lifestyle is fundamental to blood pressure control and to lowering cardiovascular risk.^6^ However, Black Americans face unique challenges in adopting and adhering to recommended self-management behaviors. Challenges include experiencing discrimination in health care settings, leading to distrust in clinicians.^8^ Interventions aimed at supporting hypertension self-management for Black veterans must address their unique experiences and challenges.

Peer narratives can address shared experiences, offering support that resonates with recipients.^9^ Trials incorporating narratives have demonstrated improvements in chronic disease prevention and management,^10,11,12,13^ blood pressure control, and hypertension self-management behaviors for individuals experiencing disparities. Previous work has studied video-recorded stories in which Black veterans with uncontrolled hypertension described their hypertension self-management.^14,15^ Although those studies found a substantial impact of the intervention on intention to change behaviors and modest effects on blood pressure, blood pressure improvements did not reach statistical significance at 6 months, suggesting the need for an extended intervention.

Ongoing support through text messages improves hypertension self-management via educational, reminder, and motivational content^16^; however, narrative text–based content has not been studied as an intervention to improve hypertension control. In this randomized clinical trial (RCT), we examined the effect of a narrative-informed texting intervention with educational content and bidirectional text messaging on blood pressure and hypertension self-management. An active control arm (bidirectional text messaging alone) was chosen to address our hypothesis that, beyond the effect of text messages, peer stories improve self-management behaviors and blood pressure.

## Methods

### Study Design

We conducted a nonblinded 2-arm multisite RCT at 2 US Department of Veterans Affairs (VA) medical centers.^14,17^ Applying within-site randomization, we assigned veterans to either the Continuing the Conversation intervention arm or the bidirectional text messages (BTMs) alone control arm. We obtained a Health Insurance Portability and Accountability Act waiver from the VA Central Institutional Review Board to identify potential participants. Initially approved in 2019, the VA Central Institutional Review Board approved modifications for an entirely virtual trial protocol in 2020 (Supplement 1). All participants provided oral informed consent. This study follows the Consolidated Standards of Reporting Trials (CONSORT) reporting guideline.

### Participants

We recruited Black veterans with hypertension from a VA medical center in Chicago, Illinois, and in Philadelphia, Pennsylvania. We oversampled female veterans to ensure adequate representation. Inclusion criteria were a diagnosis of hypertension (based on *International Statistical Classification of Diseases and Related Health Problems, Tenth Revision* codes), receipt of care at the recruiting site for 1 year or more, and 2 or more visits in the year prior to enrollment. Veterans were identified in the electronic health record (EHR) as Black or African American (hereafter Black) and taking at least 1 medication for hypertension (both confirmed via self-report). Eligible veterans had access to a cell phone, the internet, and a device for watching videos. We excluded veterans who participated in a previous video-based study^15^ and veterans who did not pass a memory and concentration screening. Pregnant veterans were excluded due to differences in hypertension management. Each potentially eligible veteran was sent an invitation letter with opt-out card. Screening telephone calls involved reviewing these inclusion criteria and collecting demographic data (Table 1).

**Table 1.** Demographic Characteristics of Black Veterans in Study (N = 600)

Characteristic	Veterans, No. (%)		
	Intervention arm	Control arm	All
Age, y			
30-49	18 (6.3)	13 (4.3)	31 (5.3)
50-69	184 (64.1)	196 (65.6)	380 (64.8)
≥70	85 (29.6)	90 (30.1)	175 (29.9)
Total	287 (100)	299 (100)	586 (100)
Sex			
Female	69 (23.2)	58 (19.5)	127 (21.3)
Male	229 (76.8)	240 (80.5)	469 (78.7)
Total	298 (100)	298 (100)	596 (100)
Educational level			
Some high school	9 (3.1)	8 (2.7)	17 (2.9)
High school diploma	77 (26.4)	83 (27.7)	160 (27.0)
Some college or technical school	125 (42.8)	136 (45.3)	261 (44.1)
College degree	41 (14.0)	43 (14.3)	84 (14.2)
Postgraduate training	40 (13.7)	30 (10.0)	70 (11.8)
Total	292 (100)	300 (100)	592 (100)
Difficulty paying for basic necessities			
Very hard	12 (4.2)	17 (5.8)	29 (5.0)
Hard	21 (7.3)	28 (9.5)	49 (8.4)
Somewhat hard	97 (33.7)	80 (27.2)	177 (30.4)
Not very hard	158 (54.9)	169 (57.5)	327 (56.2)
Total	288 (100)	294 (100)	582 (100)
Difficulty paying for medical care			
Very hard	17 (5.9)	12 (4.1)	29 (5.0)
Hard	23 (8.0)	27 (9.2)	50 (8.6)
Somewhat hard	41 (14.3)	46 (15.8)	87 (15.0)
Not very hard	206 (71.8)	207 (70.9)	413 (71.3)
Total	287 (100)	292 (100)	579 (100)
Combined family income, $			
≤15 000	43 (19.1)	32 (14.3)	75 (16.7)
15 001-20 000	26 (11.6)	26 (11.6)	52 (11.6)
20 001-40 000	52 (23.1)	58 (25.9)	110 (24.5)
40 001-60 000	47 (20.9)	45 (20.1)	92 (20.5)
60 001-80 000	29 (12.9)	29 (12.9)	58 (12.9)
≥80 001	28 (12.4)	34 (15.2)	62 (13.8)
Total	225 (100)	224 (100)	449 (100)

We calculated power to achieve a difference-in-differences (DID) in blood pressure of 5 mm Hg. We used a repeated-measures power calculation to estimate the group-time interaction for the DID analysis. Based on past work,^14,15^ we assumed 85% follow-up, a correlation of 0.5, and an SD of 18. With 600 veterans, we have more than 80% power to detect a difference of 5 mm Hg.

### Enrollment Procedures

If veterans did not own an automatic, VA-issued blood pressure monitor, one was provided to them. Once staff confirmed access to a monitor, veterans were scheduled for a telephone visit. After responding to the baseline questionnaire and taking their own blood pressure, veterans were enrolled in the VA’s Annie text messaging system^18^ and then randomly assigned to 1 of 2 arms: intervention or control (Figure). Veterans received $30 incentives per telephone visit. Recruitment occurred from March 2021 to July 2022; follow-up concluded May 2023.

**Figure.**
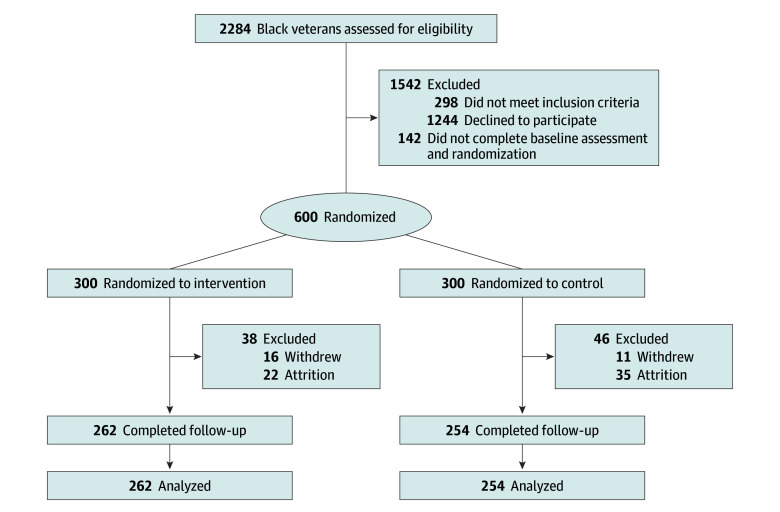
CONSORT Diagram

### Intervention and Control Arms

In the intervention arm, participants viewed video stories, selected their favorite storyteller, and then received 3 types of text messages over 6 months. We used 5 videos (5-7 minutes each) from a previous study^19^ in which Black veterans shared stories describing their personal journey of hypertension self-management. After randomization, veterans in the intervention arm were sent video links. Study staff stayed connected with the veteran on the telephone while the veteran viewed the video, or staff were called back directly after video viewing. After viewing all videos, veterans selected the storyteller whom they found most compelling, guiding which narrative text messages they received.

Our approach was informed by narrative communication theory and social cognitive theory.^20,21,22,23,24,25,26,27,28^ We hypothesized that peer stories could promote participants’ emotional engagement with the messages via a parasocial relation (ie, affective connection grounded in notions of friendship, similarity, and empathy) with the storyteller.^29^ We further hypothesized that the opportunity to select the story with greatest personal relevance, informing subsequent text messages, would enhance self-efficacy and thereby influence health behaviors.^30,31^

We identified 9 key concepts in hypertension self-management (eTable 1 in Supplement 2).^30^ These concepts guided the development of 3 distinct text message types: narrative (1-way), educational (1-way), and bidirectional (2-way). Veterans could opt-out of receiving text messages at any time.

Narrative text messages (3 per week, sent in alternating weeks) incorporated quotations and themes derived from the veteran transcripts (eg, “Willie says-basically I don’t have any plain salt in my house. Salt substitute or seasoning keeps me from craving for regular salt”). Development of text messages was informed by input from 2 Black veteran consultants, who advised on the cultural sensitivity and relevance of the messages. Methods for narrative text message development have been described previously^30^; eTable 2 in Supplement 2 provides a text message protocol example.

Educational text messages (3 per week, sent in alternating weeks) incorporated content from the American Heart Association, the American College of Cardiology, and the Centers for Disease Control and Prevention.^32^ Each educational text message focused on 1 key content area (eg, salt intake); eTables 1 and 2 in Supplement 2 provide examples.

BTM (1-4 per week) assessed self-efficacy and behavior related to hypertension self-management: “ANNIE-BP: How confident are you that you can make low salt choices shopping & eating? Text NA1 (not confident), NA2 (somewhat), or NA3 (very) to reply.” A follow-up resource suggestion was subsequently sent (eg, “If you’d like to learn more about low salt choices, check out this info on the DASH [Dietary Approaches to Stop Hypertension] diet”).

In the control arm, participants received BTMs alone 1 to 4 times per week, along with follow-up guidance on self-management. This group did not watch the video stories. Table 2 summarizes the components provided to both the intervention and control arms.

**Table 2.** Components of the Intervention and Control Arms

Component	Intervention arm	Control arm
Videos of Black veteran storytellers	Yes	NA
6-mo texting protocol		
Start text messages (3 total)	Yes	Yes
Narrative text messages based on favorite storyteller (3 per wk on alternating wks; 34 total)^a^	Yes	NA
Educational text messages (3 per wk on alternating wks; 34 total)^a^	Yes	NA
Bidirectional text messages with follow-up content, including hyperlinks to relevant resources (1-4 per wk; 35 total)	Yes	Yes
Total number of text messages planned	106	38

Abbreviation: NA, not applicable.

^a^ For the intervention, 1 week of narrative text messages alternated with 1 week of educational text messages.

### Trial Outcomes

The primary outcome was change in systolic and diastolic blood pressure from baseline to 6-month follow-up. Secondary outcomes were self-reported medication adherence, diet, physical activity, and reactions to video stories as well as trust in physicians and the VA. We also collected information on emotional connection with the video stories, number of responses to the BTMs, perceived acceptability of the text messaging intervention, and perceived need for support in handling text messages.

Blood pressure was measured according to standardized protocols adapted from the National Institutes of Health CARDIA (Coronary Artery Risk Development in Young Adults) study.^33,34^ Veterans were instructed to sit in a quiet space for 5 minutes prior to taking their own blood pressure. Veterans took 3 measurements at baseline and follow-up, and they were instructed to immediately report these readings to study staff. The mean pressures across the 3 measurements were used for analyses.

At baseline and 6 months, all veterans responded to a questionnaire administered via telephone. Hypertension management behaviors, including smoking and alcohol use, were assessed using the H-SCALE (Hypertension Self-Care Activity Level Effects).^35^ The H-SCALE has 6 domains: medication adherence (score range: 0-21, with 21 indicating perfect adherence), low-salt diet (score range: 0-7, with ≥6 indicating low-salt diet on 6 of 7 days), physical activity (score range: 0-14, with ≥8 indicating adherence to physical activity recommendations), smoking (score range: 0-7, with 0 indicating nonsmoker), weight management (score range: 10-50, with ≥40 indicating adherence to good weight management practices), and alcohol use (score range: 0-7, with 0 indicating abstinence). We used MASES-R (Medication Adherence Self-Efficacy Scale–Revised; score range: 1-4, with 4 indicating greater medication self-efficacy)^36^ and PEPPI (Perceived Efficacy in Patient-Physician Interactions; score range: 5-25, with 25 indicating greater self-efficacy talking to physicians)^37^ to measure self-efficacy. We measured trust in physicians (5 items) and trust in VA (4 items) using scales adapted from those previously developed by a member of our team^38^; both instruments used a 10-point Likert scale (strongly disagree to strongly agree), with higher values indicating higher trust. We administered the Beliefs about Medicines Questionnaire (BMQ general [8-item scale] and BMQ specific [two 5-item scales]; score range: 1-5 [strongly disagree to strongly agree], with higher values indicating stronger agreement; implications of high scores vary).^39^ We also collected demographic data as well as information on difficulty paying for basic necessities (eg, food and home heating or cooling) and difficulty paying for medical care.

Engagement for both arms was measured by tracking responses to BTMs. We assessed (from self-report) participants’ belief that the text messages provided them with motivation to manage their hypertension as well as participants’ perception of the intervention acceptability (including perceived need for support in handling text messages). For the intervention arm, immediately after video viewing, we measured the emotional and cognitive resonance of video stories using the Transportation Scale (score range: 1-7, with high scores indicating agreement; implications of high scores vary) adapted for the video stories.^14,24^ Fidelity was calculated as the percentage of intended BTMs delivered.

### Statistical Analysis

We used DID regression models for each outcome to test the hypothesis that the intervention group would have a substantially larger improvement from baseline to follow-up. Each regression model included a dichotomous time variable (baseline or follow-up), an arm variable (intervention or control), and an interaction term for arm and time. Statistical significance was prespecified at *P* = .05 for all analyses. We conducted a subgroup analysis involving those with elevated blood pressure at baseline (systolic >140 mm Hg and/or diastolic >90 mm Hg). We used random-effects models and adjusted for correlation within veterans (we did not adjust for additional variables). In addition, we tested for patterns across the whole group (intervention plus control), adjusting for correlation within veterans.

We performed an intention-to-treat analysis based on arm assigned at randomization, and analyses were conducted using nonmissing data (primary analysis). In addition, we used a regression model to evaluate whether participants who withdrew or were lost to follow-up were missing at random. Finding that these participants did not appear to be missing at random, we opted not to perform multiple imputation to avoid introducing additional bias from unobserved characteristics. Characteristics of the 84 participants who withdrew or were lost to follow-up are reported in eTables 3 and 4 in Supplement 2. As a supplementary analysis, using index dates (6 months after the day on which text messages were supposed to start), we sought to pull from the EHR available blood pressure measurements in a time window corresponding to that used for follow-up calls (2 weeks before to 3 months after the index date). Of the 84 participants, 38 had EHR-recorded blood pressure measurements in this period. We selected blood pressure readings closest to the index date (if multiple blood pressure readings were recorded on the same day, we used the mean) and incorporated these values into the supplementary analysis (eTable 5 in Supplement 2).

Engagement was calculated as the percentage of completed responses compared with all possible opportunities to respond. Fidelity was calculated as the percentage of delivered BTMs (delivered divided by intended). All analyses were estimated using Stata/MP 17 (StataCorp LLC).

## Results

We enrolled 600 Black veterans (300 from Chicago, and 300 from Philadelphia). Of participants with complete data, 469 (78.7%) were males and 127 (21.3%) were females, with a mean (SD) age of 64.0 (9.1) years. A total of 211 of 292 participants (72.3%) in the intervention arm and 227 of 300 participants (75.7%) in the comparison arm had no college degree (Table 1).

Close to half of participants in the intervention and control arms (45.1% [130 of 288] and 42.5% [125 of 294]) reported difficulty (very hard, hard, or somewhat hard) paying for basic necessities. The mean (SD) baseline systolic blood pressure was 135 (17) mm Hg, and the mean (SD) baseline diastolic blood pressure was 81 (12) mm Hg. Missing baseline data were minimal and ranged from 1.0% to 3.5%, except for total family income, about which 25.0% of participants declined to respond. Of those enrolled, 516 (86.0%; 262 in intervention arm and 254 in control arm) completed the 6-month follow-up (Figure; eTables 3 and 4 in Supplement 2). Compared with those who completed both baseline and follow-up, participants who withdrew or were lost to follow-up were more likely to report difficult paying for basic necessities (63.8% [51 of 80] vs 40.6% [204 of 502]; *P* < .001) and difficulty paying for medical care (44.4% [36 of 81] vs 26.1% [130 of 498]; *P* < .001). Accounting for all randomized participants (including those who withdrew or were lost to follow-up), the mean (SD) number of texts received across 6 months was 97.3 (27.2) in the intervention arm and 35.1 (5.1) in the control arm.

Comparing the intervention and control arms, there was no significant difference in change between arms for systolic blood pressure (DID, −0.8 mm Hg; 95% CI, –3.9 to 2.3 mm Hg; *P* = .62) or diastolic blood pressure (DID, 0.4 mm Hg; 95% CI, −1.6 to 2.4 mm Hg; *P* = .70) (Table 3). Supplemental analysis incorporating EHR-derived blood pressure values showed consistent findings for systolic blood pressure (DID, −0.9 mm Hg; 95% CI, –3.9 to 2.1 mm Hg) and diastolic blood pressure (DID, 0.6 mm Hg; 95% CI, −1.6 to 2.6 mm Hg) (eTable 5 in Supplement 2). The mean diastolic blood pressure decreased significantly in the comparison arm only (change, −2.0 mm Hg; 95% CI, −3.4 to −0.5 mm Hg).

**Table 3.** Descriptive Statistics and Difference-in-Differences Analysis for Primary and Secondary Outcomes (N = 516)

Measure	Mean (SD) values				Adjusted DID between arms (95% CI)^a^
	Intervention arm		Control arm		
	Baseline	Follow-up	Baseline	Follow-up	
Systolic BP, mm Hg	135 (16)	132 (17)	135 (18)	133 (15)	−0.8 (−3.9 to 2.3)
Diastolic BP, mm Hg	82 (11)	80 (11)	80 (12)	78 (12)	0.4 (−1.6 to 2.4)
H-SCALE: Medication adherence score^b^	18 (4)	19 (4)	18 (4)	19 (4)	−0.2 (−1.0 to 0.6)
H-SCALE: Low-salt diet score^b^	5 (1)	6 (1)	5 (1)	5 (1)	0.1 (−0.1 to 0.2)
H-SCALE: Physical activity score^b^	6 (4)	7 (4)	6 (5)	7 (4)	0.4 (−0.5 to 1.3)
H-SCALE: Weight management score^b^	36 (8)	38 (7)	35 (8)	37 (8)	0.01 (−1.1 to 1.1)
MASES-R: Medication adherence self-efficacy score^c^	4 (0)	4 (0)	4 (0)	4 (0)	−0.01 (−0.1 to 0.1)
PEPPI: Self-efficacy score^d^	22 (4)	22 (4)	22 (4)	22 (4)	0.01 (−0.7 to 0.7)
Trust in physician score^e^	44 (8)	45 (8)	43 (9)	44 (9)	0.7 (−0.8 to 2.1)
Trust in VA score^f^	32 (8)	33 (8)	32 (8)	32 (8)	0.8 (−0.4 to 2.0)
H-SCALE: Days smoking cigarettes in past wk^b^	1 (3)	1 (2)	1 (3)	1 (2)	0.1 (−0.2 to 0.3)
H-SCALE: Days drinking alcohol in past wk^b^	1 (2)	1 (2)	1 (2)	1 (2)	0.1 (−0.1 to 0.3)

Abbreviations: BP, blood pressure; DID, difference-in-difference; H-SCALE, Hypertension Self-Care Activity Level Effects; MASES-R, Medication Adherence Self-Efficacy Scale–Revised; PEPPI, Perceived Efficacy in Patient-Physician Interactions; VA, US Department of Veterans Affairs.

^a^ Adjusted for correlation within veterans.

^b^ H-SCALE^35^ score ranges: medication adherence: 0 to 21, with 21 indicating perfect adherence; low-salt diet: 0 to 7, with 6 or higher indicating low-salt diet on 6 of 7 days; physical activity: 0 to 14, with 8 or higher indicating adherence to physical activity recommendations; smoking: 0 to 7, with 0 indicating nonsmoker; weight management (5-point Likert scale: strongly disagree to strongly agree): 10 to 50, with 40 or higher indicating adherence to good weight management practices; and alcohol use: 0 to 7, with 0 indicating abstinence.

^c^ MASES-R^36^ score range: 1 to 4, with 4 indicating greater medication self-efficacy.

^d^ PEPPI^37^ score range: 5 to 25, with 25 indicating greater self-efficacy talking to physicians.

^e^ Trust in physician^38^ score range: 1 to 10, with higher values indicating higher trust.

^f^ Trust in VA^38^ score range: 1 to 10, with higher values indicating higher trust.

There were no differences between arms for secondary outcomes (eTable 6 in Supplement 2). The change in blood pressure between the intervention and control arms for those with an elevated blood pressure at baseline (n = 117 and n = 103) was nonsignificant for systolic blood pressure (DID, 3.7 mm Hg; 95% CI, −0.4 to 7.8 mm Hg), while there was a greater decrease in diastolic blood pressure within the control group (DID, 3.7 mm Hg; 95% CI, 0.8-6.6 mm Hg).

Examining patterns over time for the entire cohort, systolic (change, −2.1 mm Hg; 95% CI, –3.7 to −0.6 mm Hg; *P* = .006) and diastolic (change, −1.8 mm Hg; 95% CI, –2.8 to −0.8 mm Hg; *P* = .001) blood pressure measurements improved significantly from baseline to 6-month follow-up, as did hypertension self-management behaviors and self-efficacy beliefs (eg, H-SCALE medication adherence score: change, 0.5 [95% CI, 0.1-0.9], *P* = .02; PEPPI self-efficacy score: change, 0.5 [95% CI, 0.1-0.8], *P* = .006) (Table 4). Supplemental analysis incorporating EHR-derived blood pressure measurements showed consistent findings for systolic (change, −1.7 mm Hg; 95% CI, –3.2 to −0.2 mm Hg) and diastolic (change, −1.6 mm Hg; 95% CI, –2.6 to −0.6 mm Hg) blood pressure values.

**Table 4.** Patterns Across Entire Cohort (N = 516)

Measure^a^	Change in value from baseline to 6-mo follow-up (95% CI)^b^
Systolic BP, mm Hg	−2.1 (−3.7 to −0.6)
Diastolic BP, mm Hg	−1.8 (−2.8 to −0.8)
H-SCALE: Medication adherence score	0.5 (0.1 to 0.9)
H-SCALE: Low-salt diet score	0.3 (0.2 to 0.4)
H-SCALE: Physical activity score	0.8 (0.4 to 1.2)
H-SCALE: Weight management score	2.4 (1.9 to 3.0)
MASES-R: Medication adherence self-efficacy score	.04 (0.0 to 0.1)
PEPPI: Self-efficacy score	0.5 (0.1 to 0.8)
Trust in physician score	0.6 (−0.1 to 1.4)
Trust in VA score	0.5 (−0.1 to 1.2)
H-SCALE: Days smoking cigarettes in past wk	−0.2 (−0.4 to −0.1)
H-SCALE: Days drinking alcohol per wk	−0.2 (−0.3 to −0.1)

Abbreviations: BP, blood pressure; H-SCALE, Hypertension Self-Care Activity Level Effects; MASES-R, Medication Adherence Self-Efficacy Scale–Revised; PEPPI, Perceived Efficacy in Patient-Physician Interactions; VA, US Department of Veterans Affairs.

^a^ For score ranges for H-SCALE, MASES-R, PEPPI, trust in physician, and trust in VA, see Table 3.

^b^ Adjusted for correlation within veterans.

Across the study population, response rate to BTMs was 57.5% (11 427 of 19 875 possible opportunities to respond). Among those who completed follow-up, the response rate was 60.7% (10 564 of 17 407 possible opportunities to respond). Response rates were similar across the intervention and control arms (56.1% and 58.8%). Most (410 of 516 [79.5%]) agreed that “text messages were helpful in motivating me to improve my health,” while 75.4% (389 of 516) liked receiving text messages and 84.1% (434 of 516) believed the number of text messages received was acceptable. Over 6 months, 86.2% (445 of 516) reported that they “always” read the text messages. Few (5.0% [26 of 516]) reported that they needed to contact study staff for help with receiving text messages.

Veterans in the intervention arm stated they could relate to the video stories and storytellers, with 87.6% (211 of 241) being cognitively engaged while watching the videos, 44.6% (108 of 242) saying the video affected them emotionally, and 81.9% (194 of 237) believing the events in the video were relevant to their everyday life (eTable 7 in Supplement 2). When asked to comment on the veteran storyteller in their selected favorite video, participants agreed or strongly agreed that the storyteller “thinks like me” (79.6% [191 of 240]) and “has values like mine” (82.1% [193 of 235]). Meanwhile, participants agreed or strongly agreed that the storyteller “comes from a background like mine” (66.7% [158 of 237]) or “is a lot like me” (69.2% [162 of 234]), and 92.5% (221 of 239) of participants stated, “I could identify with the Veteran in the video.” Treatment fidelity across both arms was 92.0% (19 875 delivered text messages of 21 600 intended over 6 months).

## Discussion

Compared with the control group, participation in the intervention group did not yield additional improvement in blood pressure or secondary outcomes. The intervention was well received by veterans, who reported high rates of homophily with the video storytellers (ie, feeling emotionally engaged and believing that the storytellers had similar backgrounds and values). This latter finding is consistent with reports in prior studies of narrative storytelling.^14,15,40^

There are several possible explanations for our findings. We may have minimized the difference between arms through active engagement with the comparison arm (which received 1-4 BTMs per week with follow-up text messages linking to self-management resources). BTMs received by all participants may explain the observed significant cohort improvements in blood pressure. We may have enrolled a population already motivated to pursue healthy behaviors. Our inclusion criteria required engagement in VA care over the prior year, potentially selecting for a population more committed to monitoring their health or with greater trust in VA care. At baseline for both groups, mean home systolic blood pressure measurements met criteria for stage 1 hypertension but were not markedly elevated. While the intervention addressed communication with clinicians, this may not have translated into changes in clinician prescribing due to therapeutic inertia; assessing medication changes was outside our study scope.

Our findings are consistent with those of recently published studies. These studies, focused on texting interventions serving socioeconomically vulnerable populations with hypertension in the US and Canada (including individuals receiving care at safety net hospitals and those self-identifying as Black, Latinx, or Canadian First Nations people), also did not observe significant improvements in blood pressure.^41,42,43^ These studies varied in the frequency of outreach (daily vs weekly or several times per week)^42^; prompt content (blood pressure monitoring reminders,^42^ health behavior and medication adherence prompts,^42^ and advice on managing blood pressure and talking to clinician); and approaches to pairing text messages with clinical,^41,42^ community^43^ health worker, or social or caregiver support.^44^ As with the populations in other studies, the veterans in the present study encountered barriers to care related to social determinants of health that were likely important factors (nearly 50% of our participants reported some difficulty paying for basic necessities). The BTMs provided both groups with information on accessing resources (VA and non-VA), potentially minimizing any between-group difference. Furthermore, even if the intervention inspired participants to commit to healthier food and lifestyle choices, limited finances may have restricted their ability to do so, a challenge that these veterans may share with other socioeconomically vulnerable populations.

Favorable reactions to the health-supporting messages and high rates of longitudinal engagement were notable and may support our hypothesis that peer stories promote emotional engagement through enhancement of affective connections. Additional study is needed, however, to translate this engagement opportunity into improved clinical outcomes.

### Limitations

This trial has several limitations. First, home blood pressure checks may have decreased the accuracy of outcome measurements; however, baseline and follow-up measurements were done while on the telephone with research staff, increasing the likelihood of measure comparability. Second, we could not observe whether veterans viewed all of the videos or whether they experienced distractions. Incomplete viewing could explain a weaker effect than that of previous video-based studies, where viewing was directly observed, although the present design was more pragmatic. Finally, the study was conducted during the COVID-19 pandemic, possibly influencing veterans’ behaviors. However, the COVID-19 pandemic was marked by increased sedentary behavior, weight gain, and worsening overall health. In an isolating time when many people were foregoing health care visits for hypertension,^45,46,47,48^ receipt of text messages conveying support for self-management may have encouraged adoption and maintenance of healthy behaviors across both arms.

## Conclusions

In this RCT of video stories followed by narrative, educational, and BTMs, this intervention did not improve 6-month blood pressure compared with the control group receiving BTMs alone. Text messages supported participant engagement and enhanced motivation, and both arms demonstrated improvements in blood pressure at a time (COVID-19 pandemic) when people in the US, and disproportionately Black veterans, were experiencing worsening hypertension control.^45,46,47,48^ Text messaging for hypertension self-management, embedded within a virtual delivery strategy, can support engagement and motivation for Black veterans with hypertension.

## Notes

Supplement 1.
Trial Protocol

Supplement 2. **eTable 1.** Key Hypertension Self-Management Content Areas Incorporated Into Texting Protocols for Intervention and Control Group **eTable 2.** Example of the First 9 Weeks of Text Message Protocol for a Single Veteran Storyteller **eTable 3.** Demographic Characteristics: Comparison of Participants Completing Study to Those With Missing Follow-Up Data (Withdrawn or Lost to Follow-Up) **eTable 4.** Demographic Characteristics of Participants Missing Follow-Up Data (Withdrawn or Lost to Follow-Up) **eTable 5.** Supplemental Analysis Incorporating EHR-Derived Follow-Up Blood Pressure for Participants Missing or Lost to Follow-Up **eTable 6.** Descriptive Statistics and Difference-in-Differences Analysis for Additional Outcomes of ‘Continuing the Conversation’ (CTC) Randomized Controlled Trial **eTable 7.** Experiences and Reactions to Watching Video-taped Veteran Stories About Hypertension Self-Management

Supplement 3.
Data Sharing Statement
