# Supplementary material for: Text Messaging and Video Stories to Support Hypertension Self-Management in Black Veterans: A Randomized Clinical Trial
Source: JAMA Netw Open. 2025 Nov 5;8(11):e2541342. doi: 10.1001/jamanetworkopen.2025.41342 (PMC12590299; doi:10.1001/jamanetworkopen.2025.41342)
Supplement: Supplement 1. — Trial Protocol [file jamanetwopen-e2541342-s001.pdf]

17-185 *“Continuing the Conversation: A Multi-site RCT Using Narrative Communication to Support Hypertension Self-Management among African-American Veterans”*

Funding Agency: HSR&D

Principal Investigator/Study Chair: Sarah Cutrona, MD MPH

February 10, 2022 / Version 7

VA Central IRB
Effective Date: June 6, 2022

Abstract

Background: Compared to other racial and ethnic groups, non-Hispanic African Americans have an earlier onset and higher prevalence of hypertension (HTN) as well as a disproportionately higher risk of complications, leading to higher rates of cardiovascular morbidity and mortality. In previous work, we used storytelling in African Americans outside the VA, yielding significant improvements in blood pressure. We translated these findings to the VA, developing and testing video-recorded Veteran narratives which we showed to African-American Veterans. We demonstrated significant differences in intention to change HTN management behavior immediately after video viewing; however, effects on blood pressure were not sustained, and six-month outcomes revealed only modest benefit over control ($p = 0.06$). Our findings highlight the need for longitudinal support to sustain the storytelling effect. Our proposed study provides longitudinal support via text messages, incorporating content from the participant's chosen peer narrative as a means of sustaining motivation and engagement in HTN self-management.

Innovativeness: Our study, "Continuing the Conversation," is a novel integration of peer narrative communication into technology. While use of an informatics tools (texting) as a channel to support self-management is not novel per se, the extension of a narrative via longitudinal texting is an innovative mechanism for supporting and sustaining HTN self-management behaviors. Our **Specific aims include**:

Aim 1. Refine and Pilot the Continuing the Conversation (CTC) intervention. Previously, we created video-recorded stories told by African-American Veterans with HTN, describing their self-management strategies. We will refine CTC by adapting content from these videos to create narrative-aligned texts and we will pilot CTC with 20 Veterans.

Aim 2. Test CTC by conducting a randomized controlled trial. CTC 'begins the conversation' by showing Veteran Story videos to participants, then inviting participants to select a preferred narrative. We then 'continue the conversation,' offering longitudinal support via 6 months of narrative-aligned text messages. Messages cover key HTN content, providing education, reminders and weekly assessments, and include quotations derived from the chosen narrative. Control participants receive weekly assessment texts addressing the same key HTN self-management behaviors.

Aim 3. Evaluate CTC effectiveness, and mediating factors, and conduct a cost analysis. We hypothesize that, for the CTC Intervention group as compared to the control: **(H1)** the difference in blood pressure from baseline to 6 months (primary outcome) will favor CTC intervention compared with the change in control. (**H2**): Self-efficacy and HTN management behaviors during 6-month follow-up will be greater for those in the CTC intervention group than control.

Methods: In a randomized controlled trial, 600 African-American Veterans with HTN will be recruited from 2 VA healthcare sites with known disparities in HTN control. We will use within-site randomization (CTC vs. Control). Outcomes will include blood pressure, self-efficacy and HTN management behaviors. Longitudinal texts are designed to bring the storyteller back into the Veteran's everyday life, reminding and reinforcing as our Veterans engage in the numerous daily decisions that will impact their blood pressure and their lives. Incorporating peer content into text messages in this way is highly innovative and offers a promising approach to supporting our Veterans.

VA Central IRB
Effective Date: June 6, 2022

List of Abbreviations

Provide a list of all abbreviations used in the protocol and their associated meanings.

PI/SC: Principal Investigator/Study Coordinator

HIPAA: American Health Insurance Portability and Accountability Act

SAE: Serious Adverse Event

LSI: Local Site Investigator

HTN: Hypertension

RA: Research Assistant

VA Central IRB
Effective Date: June 6, 2022

Contents

Protocol Title: 5
1.0 Study Personnel 5
2.0 Introduction 6
3.0 Objectives 7
4.0 Resources and Personnel 7
5.0 Study Procedures 8
5.1 Study Design 8
5.2 Recruitment Methods 13
5.3 Informed Consent Procedures 14
5.4 Inclusion/Exclusion Criteria 14
5.5 Study Evaluations 14
5.6 Data Analysis 16
5.7 Withdrawal of Subjects 16
6.0 Reporting 17
7.0 Privacy and Confidentiality 17
8.0 Communication Plan 17
9.0 References 18

VA Central IRB
Effective Date: June 6, 2022

Protocol Title: "Continuing the Conversation: A Multi-site RCT Using Narrative Communication to Support Hypertension Self-Management among African-American Veterans"

1.0 Study Personnel

- Provide name, contact information, and affiliations/employee status for the following:

Principal Investigator/Study Chair:

- Sarah Cutrona, MD, 781-687-2861, sarah.cutrona@va.gov, 8/8ths VA Employee VA Bedford Healthcare System

Co-Investigators:

- Barbara Bokhour, PhD, 781-687-2862, Barbara.bokhour@va.gov, 8/8ths VA Employee VA Bedford Healthcare System
- Stephanie Shimada, PhD, 781-687-2208, stephanie.shimada@va.gov, 8/8ths VA Employee VA Bedford Healthcare System
- Gemmae Fix, PhD, 781-687-3368, gemmae.fix@va.gov, 8/8ths VA Employee VA Bedford Healthcare System
- Judith Long, MD, 215-823-5800 x5875, Judith.long@va.gov, 8/8ths VA Employee Corporal Michael J. Cresenz VAMC
- Howard Gordon, MD, 312 569-7473, howard.gordon2@va.gov, 8/8ths VA Employee Jesse Brown VAMC
- Charlene Pope, PhD, 843-789-6577, Charlene.pope@va.gov, 8/8ths VA Employee Charleston VAMC
- Bridget Smith, PhD, 708-202-8387 x24870, bridget.smith@va.gov, 8/8ths VA Employee, Hines VAMC
- Kevin Stroupe, PhD, 708-202-8387 x23557, kevin.stroupe@va.gov, 8/8ths VA Employee Hines VAMC

VA Central IRB
Effective Date: June 6, 2022

2.0 Introduction

- Hypertension (HTN) and complications of hypertension are unusually common among African Americans, leading to higher rates of cardiovascular morbidity and mortality. According to the National Health and Nutrition Examination Survey 2011-2014, the prevalence of HTN was highest among non-Hispanic African-American/black adults (42% for men; 46% for women) compared to non-Hispanic whites (31% men/30% women), Hispanics (27%/32%) and Asians (29%/27%).[1] Disparities in HTN control may derive from differential rates of diagnosis, treatment titration, and patient factors including medication adherence, lifestyle changes, self-monitoring, and engagement with the healthcare system. African-American patients routinely report lower trust in the healthcare system,[2] which may limit their willingness to adhere to doctor-recommended treatments[3]. Effective HTN control through lifestyle change and medication adherence entails numerous small, repeated decisions. Peer interventions can help to counter cognitive resistance which arises when patients have concerns about treatments or about medical information. Narratives are a widely accepted and effective way to engage listeners and reduce cognitive resistance.[4-8] Narrative communication (storytelling) has been defined as “any representation of a sequence of connected events and characters that has an identifiable structure, is bounded in space and time, and contains implicit or explicit messages about the topic addressed.”[9] The effectiveness of narrative communication in changing attitudes and behavior follows from transportation and personal identification.[10] Findings from our previous studies support the hypothesis that Veteran peer narratives will have a greater and more sustained impact if followed by longitudinal communications that ‘continue the conversation’. This project addresses a key issue – HTN control in African Americans. We believe it is important to pursue both traditional and novel dissemination strategies to share lessons learned. If we find the intervention to be successful, we will create implementation facilitation tools including an Integrated CTC intervention guide (hyperlinks to storyteller videos, electronic downloadable copies of Veteran-facing materials including Storyteller Selection cards, orientation brochures for the texting system, training guides for staff). We will work with OCC to create an Electronic Health Record-based referral tool to encourage teams to enroll patients in CTC and will explore mechanisms by which Veterans can initiate enrollment requests. We will disseminate findings from our work at health behavior change venues, including the Society of Behavioral Medicine, as well as at health informatics venues such as American Medical Informatics Association Annual Meeting. As this work will ultimately be most effective if it is taken up by clinical practitioners and by the Veterans themselves, we will also seek to reach clinical audiences including those at Society for General Internal Medicine and will work with our Veteran Advisors as well as with our local and national Veteran’s Engagement Workgroups to disseminate information on the CTC intervention through channels accessible to and frequented by Veterans.
- Our intervention is intended for African-American Veterans, and we will intentionally oversample women Veterans with the goal of including in the sample 20% women (60 women at each site, 120 total). Children and prisoners will not be included in this study, as those under the age of 18 are not qualified and it is not feasible to include prisoners in the study.

3.0 Objectives

VA Central IRB
Effective Date: June 6, 2022

Our **Specific Aims** include:
Aim 1. **Refine and pilot the Continuing the Conversation (CTC) intervention**.

- **1a**. With input from Veteran Advisors, we will refine our CTC intervention, adapting content from our previously developed online videos (stories told by African-American Veterans who have controlled their hypertension) to create narrative-aligned text messages (‘storyteller texts’).
- **1b**. We will pilot the process of providing guidance to 20 Veterans as they (a) **View** Veteran videos; (b) **Select** a preferred video narrative; (c) Sign up to **Get** texts through HealtheDialog Texting system.

Aim 2. Test CTC by conducting a randomized controlled trial with the following characteristics:

- <u>Setting and Sample</u>: 600 African-American Veterans with hypertension. Veterans will be recruited from two VA healthcare sites with known disparities in hypertension control.
- <u>Design</u>: Veteran-level randomization, stratified by site and within-site stratification by gender
- <u>CTC Intervention</u>: An integrated peer narrative-based intervention (VIEW > SELECT > GET), beginning with VIEWing of VA Stories narratives, followed by SELECT a favorite Veteran Storyteller, and then GET favorite Storyteller “narrative-aligned” text messages over 6 months
- <u>Control</u>: 6-month HTN management assessment text messages without narrative component

Aim 3. Evaluate CTC effectiveness, and mediating factors, and conduct a cost analysis.

- We hypothesize that, for those in the CTC Intervention group as compared to the control:
- **<u>Hypothesis 1</u>**: The difference in blood pressure from baseline to 6 months (primary outcome) will favor CTC intervention group by 5mm Hg, compared with the change in control.
- **<u>Hypothesis 2</u>**: Self-efficacy and hypertension management behaviors during 6-month follow-up will be greater for those in the CTC intervention group than comparison.
- We hypothesize that (**H3)** the effect of CTC on blood pressure will be mediated by the proximal measures of **(H3a)** cognitive and emotional processes (i.e.: emotional engagement with stories, self-efficacy) and **(H3b)** hypertension self-management behaviors. We will further measure the marginal cost of this intervention.

4.0 Resources and Personnel

- Led by Principal Investigator and Co-Investigators at the Bedford VA, two sites will be recruiting patients for this study under the supervision of LSI’s at each site: Corporal Michael J. Crescenz VA in Philadelphia, under the supervision of Judith Long, MD and Jesse Brown VA in Chicago, under the supervision of Howard Gordon, MD.
- Dr. Cutrona will be Principal Investigator on the study. Dr. Cutrona will oversee and coordinate activities carried out by the **Text Content Development Team** (Drs. Bokhour, Fix, Pope, Veteran Advisors), the **Texting Protocol Development &**

VA Central IRB
Effective Date: June 6, 2022

Implementation Team (Dr. Shimada, Veteran Advisors) and the **Pilot/Trial Team** (Drs. Long, Gordon, Veteran Advisors). The Content Team will adapt content for text messages from existing video and interview transcripts. The Protocol Team will adapt existing HealtheDialog texting protocols for use in the CTC intervention, will create brochures for Veterans with information about the texting system and will also develop guides for RAs (how to enroll Veterans in text system). Drs. Long and Gordon will oversee the pilot intervention at their respective facilities and the trial in subsequent years. **Veteran Advisors** will provide focused input on content and acceptability of text messages and laminated video selection cards; timing and enrollment procedures for texting protocol; and acceptability of recruitment material. Advisors**,** including Dr. Timothy Hogan, Director of the eHealth Partnered Initiative at the Center for Healthcare Organization and Implementation Research (CHOIR), Drs. Jeroan Allison, and Thomas Houston will be consulted regularly, providing content area expertise and input as needed on intervention development and implementation. Dr. Smith will lead the analytic plan and cost analysis for the project, Dr. Stroupe will contribute to the cost analysis for this project. Zhiping Huo will assist with data management under the direction of Drs. Smith and Stroupe.

5.0 Study Procedures

5.1 Study Design

- <u>Design for Aim 1: Refine and Pilot</u>: We will begin by refining and piloting the Continuing the Conversation (CTC) intervention with 20 Veterans. We will adapt content from our previously developed online videos (stories told by African American Veterans who have controlled their hypertension) to create narrative-aligned text messages (‘storyteller texts’). Interviewees have already provided consent to use content from their interviews beyond the previous study. Transcripts from previous interviews will be used to inform content for these text messages.
- We will pilot the process of providing over-the-phone guidance to Veterans as they (a) **View** the Veteran videos; (b) **Select** a preferred video narrative; and (c) Sign up to **Get** texts through the HealtheDialog Texting system. The pilot will contain usability testing and a formative evaluation of the Veterans’ experiences. We will purposefully sample 10 Veterans from each site (total of 20) and will aim to include 4 women Veterans (2 per site). We will pilot the process of (1) guiding the Veteran in at-home BP check, (2) selection of a preferred veteran storyteller after viewing all stories; (3) using this choice to inform selection of a texting protocol; (4) Receipt of 1 month of texts; and (5) follow-up assessment at the end of 1 month. We will assess feasibility of two options for Veterans to access texts: (a) via regular texting, available on all text-enabled phones; (b) via HealtheDialog ‘App’ which allows study team tracking of text opening (but is available only on Smart phones). We will assess completion rates (how many Veterans have difficulty or discontinue texts early, reasons why) and perceptions (e.g. "I felt connected to the storyteller while receiving texts", "the number of texts was: too few, just right, too many”). The formative evaluation will include a qualitative interview with all Veteran participants to understand their experiences with the CTC protocol. Our semi-structured interviews will cover their overall experiences, as well as specific questions about the enrollment process, frequency and content of messages. RA’s will take brief, descriptive field notes.

VA Central IRB
Effective Date: June 6, 2022

- We will then Test CTC by conducting a randomized controlled trial with the following characteristics:
- Setting and Sample: 600 African-American Veterans with hypertension. Veterans will be recruited from two VA healthcare sites with known disparities in hypertension control.
- Design: Within-site randomization of Veterans
- CTC Intervention: An integrated peer narrative-based intervention (VIEW > SELECT > GET), aligned with the process of providing over-the-phone guidance to Veterans as they (a) **View** the Veteran videos; (b) **Select** a preferred video narrative; and (c) Sign up to **Get** 6-months of narrative, educational and assessment texts through the HealtheDialog Texting system. We will be guiding the Veteran in (1) at-home BP check, (2) selection of a preferred veteran storyteller after viewing all stories; (3) using this choice to inform selection of a texting protocol; (4) Receipt of 1 month of texts; and (5) follow-up assessment at the end of 6 months.
- Control: 6-month VA HTN texting protocol without narrative component
- Blood pressure will be checked with guidance from the RA over-the phone using an at home BP monitor at baseline and at a 6-month follow up for both arms.

HealtheDialog Texting system, Sending and Receiving Texts

- The Office of Connected Care (OCC), with whom we have partnered, has invested in HealtheDialog, an automated text messaging system recently developed and field-tested in five VA facilities. Text messages are automatically sent from the pre-programmed automated VA text messaging system to users. Veteran participants will receive text messages delivered using this VA HealtheDialog texting system.

- Protocols for text messaging will be developed in year 1 based on existing hypertension protocols (for further details see 'CTC Texting Protocol' below and Appendices 3-6). Details are provided below; briefly, for the intervention group, weekly texts will include up to 3 narrative-aligned texts (messages that reinforce the voice and themes brought up in videos regarding HTN self-management, 'e.g. Danny says: 'I try to take my med at the same time every morning') and 1 assessment text (e.g. 'On how many of the past 7 days did you take your BP pills at the same time every day? Text 1 thru 7). Intermittently, a week will include no narrative texts and up to 4 assessment texts. Control participants will receive 1 assessment text weekly. For assessment texts, users send a keyword response based on the instructions in the previous message, e.g., "Did you take your medications today? Reply: MED Yes or MED No." Depending on if the patient responded "MED Yes or MED No," HealtheDialog automatically sends a programmed response (e.g., "Great work!"). Incoming and outgoing messages activity logs can be viewed and recorded through the system dashboard.

Time and Days of Text Delivery

- While the protocols allow time of day to be adjusted for each participant, leadership of the HealtheDialog system do not currently recommend this approach due to the level of complication and resulting burden that it adds to the automated system. Such modifications will also increase the time required for enrollment since (in the system as currently configured) the person signing up the patient (the RA) must go in and manually change each message's delivery time. We recognize that for a longitudinal texting support program to be most useful to our Veterans, it would be optimal to have some ability to customize timing. In our year 1 development phase, we will work closely with the Office of Connected Care to identify options to allow flexibility on timing, and during our pilot phase we will aim to explore feasibility of customizing timing at enrollment by

VA Central IRB
Effective Date: June 6, 2022

assigning messages to be sent at a few different times and obtain feedback during our pilot follow-up specifically regarding participant thoughts on times texts were received.

Videos, Procedures surrounding Video viewing and Selection of text protocol: Our team has already created and tested video-recordings available online (Appendix 1) in which African-American Veterans with controlled HTN share their stories (describing challenges and successes managing their HTN).

- Procedure for Intervention group participants.

VIEW videos: During the over the phone visit, the Research Assistant (RA) will ensure the participant has a quiet space and online access to the 5 Veteran narratives so that participants can view the 5 brief videos. We will send the link to the videos to the participant if they have given permission to contact via email, otherwise we will send them the link via text, or read it aloud to them. Veterans will not be provided DVDs but since they will be provided with the web address they can access the videos on their own if they wish to re-view videos. If the Veteran requests use of a virtual platform to watch the videos, such as WebEx, Zoom, or other approved platform, the RA will create a session and utilize the shared screen to watch the videos only. No PHI or PII will be shared during this time.

SELECT a Preferred Veteran Storyteller: After the Veteran has viewed the videos, the Veteran will complete the final brief section of the survey. If necessary the RA will re-contact via phone or by text at that time to ask survey questions. The RA will remind participant of the five storytellers they just watched. If at that time the Veterans wish to pause and go back to see any videos they missed or re-view any videos, they may. The RA will explain that this selection will inform the content of the text messages sent to the Veteran over the upcoming 6 months, with the goal of providing text messages that work well for the Veteran. The RA will explain that some messages will include either (a) direct quotes from this storyteller, referencing back to or building further upon the video-recorded story; or (b) references to topics related to those which the Veteran talked about. If **participant is not reached following watching the storyteller videos, the site coordinators will initiate a text requesting that they select a storyteller. If they do not reply to the text request and are still unreachable 2-weeks after the baseline visit occurs, we will select a storyteller for them by selecting, in order,from a list of the 5 storytellers; when all 5 have been assigned in this way, we will start back at the top of the list. Lists will be maintained separately at the two sites. Participants will be told during the baseline visit, once assigned to intervention, after watching the videos that if they do not select a storyteller for some reason, they will be assigned one by the research team.**

GET preference-based, narrative-aligned CTC texts: Veteran participants will receive text messages, with messages chosen to align with their selected storyteller. These texts will be delivered using the VA HealtheDialog texting system, described above. In the baseline phone visit, Veterans will receive an orientation to the texting system, over the phone. RAs will enroll Veterans in HealtheDialog and provide education on use of the system and what to expect from the CTC protocol.

- Procedure for Control Group Participants:

VA Central IRB
Effective Date: June 6, 2022

Veterans will have an over-the-phone visit during which the RA enrolls each Veteran in the control texting protocol and collects baseline data including blood pressure and all Baseline elements described in Section 5.5. Veterans will then receive 6 months of text messages, receiving one assessment text weekly with a follow-up reminder if needed, and they will also receive responses to their reply to assessment texts.

CTC Texting Protocol (content and tasks required from participant):

- Narrative-aligned Texts (“Storyteller Texts”): Participants will be invited to choose the Veteran storyteller whom they found most compelling; this choice will then be used to guide selection of a longitudinal texting protocol designed to ‘continue the conversation’ over a six-month period. Storyteller texts are intended as cues, triggering recollection of the foundation laid through the initial story viewing. Our intention is to use these texts to engage participants on both an emotional and cognitive level. We will adapt transcribed content from our VA Stories videos to create narrative-aligned texts (“storyteller texts”) (Appendix 3). Adaptation of transcribed content will be conducted with 3 goals: to identify quotations for which (1) the clarity and length are conducive to incorporation into a stand-alone text message, (2) content maps to our conceptual model and (3) to ensure that quotations convey the storyteller’s ‘voice’ (their own words/phrases). Where direct quotations conducive to incorporation into text are not available, narrative-aligned texts will convey messages that reference and build upon the story content.

- Our team has already completed qualitative analyses of full (unedited) video recordings of Veteran narrators. Completed analyses identified story segments guided by storytelling best practices and informed by constructs from our conceptual model; this work will inform our selection of content for storyteller texts.

- In year 1, we will create HealtheDialog texting protocols corresponding to each of the video-recorded Veteran storytellers. To create the CTC texting protocol (Appendix 4), we will adapt existing texting protocols. We will include core content via the longitudinal texting protocols that is consistent across texting protocol options. Key concepts (such as talking with your doctor, taking your medications, avoiding salt and physical activity) will be delivered to all participants, with messages customized to reflect the voice of the selected storyteller as detailed above.

- To create these protocols, we will adapt content from unedited videos to create narrative-aligned text messages (‘Storyteller texts’). In our original storytelling intervention, we used EHR data to identify African-American Veterans who had successfully controlled their HTN. From this group, potential Storytellers participated in screening interviews and were selected based on ability to tell engaging, authentic stories about HTN management behaviors. For each selected storyteller, the research team facilitated an hour-long, videotaped “story” in which the Veteran described successfully controlling his/her HTN. These stories were qualitatively coded to identify potential video content. Next, potential content was systematically ranked by Veteran and research team members. This process culminated in short videos by the 5 “best” storytellers. In our prior study, we obtained approvals from each participant to use content of the videos and transcripts in education and research materials beyond the last Stories project period. We obtained all appropriate waivers and permissions for further use of recorded audio and videos; these remain available as outlined in the Data Inventory Tool, on file with the Information Security Office in Bedford.

VA Central IRB
Effective Date: June 6, 2022

- We will also create an **assessment protocol** which will send assessment texts to the Veteran once a week (for example, asking if they have missed any HTN medications in the past week, YES/NO response).

- To validate proper programming of the protocols into the system, we will enroll CPRS test patients as ‘participants’ to receive texts and ensure the correct texts are delivered at the correct times.

- Intervention participants will receive up to sixteen text messages weekly for the duration of the 6-month study. Themes will address our HTN management behaviors and will also include general motivational statements. Included in the protocol will be texts designed to elicit a Veteran response to a question assessing status of a specific HTN self-management activity. These assessment texts will be adapted from existing HealtheDialog texting protocols and from the H-SCALE (Hypertension Self-Care Activity Level Effects), a set of 31 measures including point prevalence measures (in the past seven days) for low-salt diet and physical activity. Weekly texts will include up to 3 narrative-aligned texts (‘e.g. Danny says: ‘I try to take my med at the same time every morning’) and 1 assessment text (e.g. ‘On how many of the past 7 days did you take your BP pills at the same time every day? Text 1 thru 7), with a follow-up reminder text if needed. In addition, participants will receive responses to their assessment texts replies. Intermittently, a week will include no narrative texts and up to 4 assessment texts with responses.

  Phone number changes
- During enrollment into HealtheDialog, an action that will be done over-the-phone together with the RA, a pop-up screen will prompt RA to remind Veterans to tell our study team if their phone number changes. If this happened, we would update the number in the system. The HealtheDialog texting system dashboard does not collect a read receipt, but it would show if a patient doesn’t send a response message back.
- Following best practice, baseline differences between intervention and comparison groups will be based on standardized differences, not simply tests of statistical significance. All primary hypotheses will be tested by intent-to-treat; secondary analyses will account for important covariate imbalances between groups, levels of adherence to the intervention protocol, and missing data. All analyses will be two-sided, with alpha error = 0.05.
- Our randomized controlled trial will be conducted at two sites.
- The study population will consist of African-American Veterans, both male and female, with diagnosis of HTN. We anticipate recruitment of 600 African-American Veterans for this study.
- Minimizing Risk: Every effort will be made to make study participants comfortable when completing questionnaires or watching the video, and we will clearly communicate expectations regarding frequency of text messages at the time of study enrollment to avoid inconveniences. We will also provide standard safety advice for text message use (e.g. avoid reading texts while driving or walking).

VA Central IRB
Effective Date: June 6, 2022

5.2 Recruitment Methods

- For the pilot, we will purposefully sample 10 Veterans from each site (total of 20) and will aim to include 4 women Veterans (2 per site).
- For the main RCT, We will recruit 600 African-American Veterans with HTN. Veterans will be recruited from two geographically distinct VAMCs (Chicago and Philadelphia, Table 7) with a high proportion of African-American patients and known disparities in HTN control.
    - o Jesse Brown VAMC in Chicago is a 189-bed hospital and teaching affiliate of the University of Illinois-Chicago and Northwestern University. The Jesse Brown VAMC and its four affiliated community-based outpatient clinics (CBOCs) in Chicago, Southern Cook County, and northwest Indiana provide primary care to roughly 30,000 veterans and 80% are African-American. Among primary care patients, approximately 18,000 have HTN.
    - o Corporal Michael J Crescenz (CMJC) VAMC in Philadelphia serves over 90,000 Veterans, operating CBOCs in Fort Dix, NJ, Gloucester County, NJ, Horsham, PA, and Center City Philadelphia.
- Using VISTA data, we will review data for potentially eligible patients for up to 2 years preceding recruitment. We will identify patients with a listed diagnosis of HTN (ICD10 diagnosis codes: *I10, I11.0, I11.9, I12.0, I12.9, I13.0, I13.10, I13.11, I13.2, I15.0, I15.1, I15.2, I15.8 I15.9, I16.0, I16.1, I16.9, N26.2*) (further details on inclusion criteria listed below). The final generated list of potential participants will be kept in a computer file that is password protected and only accessible by the RA and project staff. Any printed list used by the RA in the clinic will be stored in a locked file cabinet when not in use. Staff will make all attempts to print the recruitment letters and labels, and stuffing the envelopes at the VA location, however if a location other than the VA is necessary for this task, all security requirements will be in place for transporting any study materials to the secondary work location of the RA's home; ie. a signed Authorization to Transport, and RA will use a locked safe to store printed materials containing PII. We will include both women and men in the study.
- Each potentially eligible Veteran will be sent a letter inviting participation, with an opt-out card. If no opt-out card is received in a two-week period, the RA will contact the Veteran by telephone, explain the study and ask several eligibility screening questions including the patient's race/ethnicity and access to a cell phone with texting capability. Competency will be assessed during the phone screen questionnaire which includes cognitive functioning questions.
- If the Veteran is eligible and wishes to participate, the RA will schedule a time for the Veteran to hold the visit over-the-phone.
- At the start of the over-the-phone study visit and at the start of the follow-up phone visit (for both pilot and RCT) the RA will confirm the Veteran's identity and confirm the address at which he or she will be located for the duration of the phone call. This will provide RAs the ability to notify emergency personnel in the event that an emergency response is needed for any reason.
- Intervention and control Veteran participants will both receive the same incentives. Each participant will receive a $30.00 incentive per phone visit, for a total of two visits (in-person baseline visit and follow-up data collection visit). Payment method may vary by site and will be based on what is available and most convenient for the Veterans.

VA Central IRB
Effective Date: June 6, 2022

Participant may incur costs related to receiving or sending texts if they do not have an unlimited texting plan; they will be notified of this in our consent form.

Potential risk to participants when receiving/responding to texts while walking/driving etc.

- During enrollment of participants, RA's will review texting safety measures including avoidance of texting and driving (we will review available 'do not disturb' phone features with Veteran as desired), avoidance of texting while walking (or doing other mobile activity that requires visual attention); ensuring physical safety before pausing to review or respond to texts.

5.3 Informed Consent Procedures

- We will be obtaining a waiver of informed consent documentation for all study activities. We will be requesting a HIPAA Waiver to obtain the participant list from which to recruit. For over-the-phone visits we are submitting a waiver of documentation of informed consent.
- We will train site RA's at both sites in procedures to follow for baseline and follow-up visits. Screening for eligibility and competency will be conducted during the phone screen questionnaire which includes cognitive functioning questions. If the participant is eligible, able to complete the competency questions and is willing to participate, a baseline visit will be scheduled over-the-phone.
- The Bedford project manager will hold weekly training sessions including using Teams when needed to review and train RA's at local sites in obtaining informed consent and conducting visits.

5.4 Inclusion/Exclusion Criteria

- We will include Veterans who have been receiving care at the recruiting VA site for ≥1 year prior to recruitment, with 2 or more visits documented over the past year. We will include Veterans who have documented HTN (ICD10 diagnosis codes: *I10, I11.0, I11.9, I12.0, I12.9, I13.0, I13.10, I13.11, I13.2, I15.0, I15.1, I15.2, I15.8 I15.9, I16.0, I16.1 I16.9, N26.2*) during this 1-year period. We will include only patients who self-identify as African American or Black. In addition, participants must be on at least one medication for BP. Eligible participants will need to have access to their own or a family member's cell phone or smart phone for participation and they must be willing to use this phone for receipt of text messages over a 6-month period. Eligible participants will also need to have access to internet and a reliable device in order to watch videos. If Veterans do not have a BP monitor at home, we will arrange to have one sent to their home – they must be willing to take their own BP or have someone assist them in doing so. We will exclude Veterans who participated in our previous VA Stories study.

VA Central IRB
Effective Date: June 6, 2022

In addition, any Veteran who fails the memory and concentration questions asked in the screening survey will be excluded. Because hypertension management guidelines for pregnant females differ, pregnant women will be excluded; we have included this in the screening survey.

5.5 Study Evaluations

- We will pilot the process of (1) guiding the Veteran in at-home BP check; (2) selection of a preferred veteran storyteller after viewing all stories; (3) using this choice to inform selection of a texting protocol; (4) Receipt of 1 month of texts; and 5) follow-up assessment at the end of 1 month. Research assistants will collect field notes on items (1) (2) and (3). For item (4) we will collect data on implementation/process measures throughout the 4-week period (see Table 8 below, item 7 for details of these measures). We will also assess feasibility of two options for Veterans to access texts: (a) via regular texting, available on all text-enabled phones; (b) via HealtheDialog 'App' which allows study team tracking of text opening (but is available only on Smart phones).
- All data will be stored in accordance with VA policy.
- Only the study team and the PI will have access to this information.

For the randomized controlled trial, as listed in the table below, we will collect 7 data elements: (1) Veteran characteristics; (2) Intervention dose/exposure; (3) Narrative Communication measures; (4) Social Cognitive-Theory-based measures; (5) Hypertension management behaviors; (6) Blood Pressure (BP) (blood pressure will be checked by participant through home BP monitor at baseline and at 6 month follow up); and (7) Implementation Data including Process Measures and measures of Fidelity. We will gather these elements through initial data collection (CDW), over-the-phone screening survey, baseline visit and follow-up visit observations and surveys, texting system and phone calls to participant.

VA Central IRB
Effective Date: June 6, 2022

• **Table 8. Seven Key Data Elements**	Timing	Source
**1.Veteran Characteristics**		
Participant Demographics	1	S,C
Comorbidities, VA Utilization	1	C
**2. Intervention Dose/Exposure**		
Number of videos watched and time spent viewing	2	S
Which was selected video?	2	S
**3. Narrative Communication**		
Video Transportation Scale	2	S
Homophily (personal relevance)	2	S
**4. Social Cognitive Theory**		
Beliefs About Medications (BMQ)	1,4	S
Self-efficacy for hypertension (e.g.: MASES-R, PEPPI)	1,4	S
**5. HTN management behaviors**		
Avoid Salt, Be Physically Active, Monitor your BP(H-SCALE, PEMH)	1,4	S
Talk with your doctor (H-SCALE)	1,4	S
Take Your Medicines: Morisky ; and administrative data adherence (MPR)	1,3,4	S, C
**6. Primary Outcome (H1)**		
Measured BP (systolic/diastolic)	1,4	BP, C
7. **Implementation Data/ Process Measures/Fidelity**	3,4	RA, S, T
Text message opening (subgroup)	3	T
Responses to text assessment	3	T
Confirmation of text receipt	4	S
Phone calls for texting assistance	3	RA
Staff time spent for implementation	3	RA
Persistence/Early Discontinuation of Texting Protocol	3	T
Reasons for discontinuation	5	P
1=Baseline, 2=Immediately post-Video Viewing; P, 3=Repeated throughout 6-month study period, 4 = six-month follow-up; 5= phone call to drop-out<br>Source: S = Survey; C = CDW data; RA=Research assistant; T=Texting System; P=phone call to participant; BP = Blood pressure cuff, standard protocol (For BP measurement at 6-months, we will pull CDW data for the entire study participation period)		

5.6 Data Analysis

With our recruited sample of 600 (and assuming follow-up rates, over-time within-person correlation, and BP standard deviations from our prior work (85% follow-up, correlation = 0.5, SD = 18 (equivalent to variance of 324)), we have over 80% power (alpha 0.05) to detect a difference of 5mm Hg. In fact, we calculated power for a range of scenarios, and are able to maintain the 5mm Hg detection with a lower follow-up rate of 82%.

Power for H2 & H3: Conceptually, the CTC intervention impact on blood pressure is possible because the intervention will have an effect on more proximal cognitive and behavioral outcomes (H2) that then mediate the main effect (H3). Thus, theoretically, power for group-by-time differences in the scales for H2 will be greater than H1, and thus we should be over-powered for these measures. We calculated H2 power for difference-in-differences analyses. As each of the scales were different, we report minimum detectable differences as effect sizes.

Dr. Cutrona will lead the data analysis in years 3 & 4, working closely with Drs. Smith and Stroupe (statistician, cost analyst) and programmer.

5.7 Withdrawal of Subjects

VA Central IRB
Effective Date: June 6, 2022

- If at any time the local site PI feels the study is inappropriate for a participant or due to medical reasons, the PI reserves the right to discontinue/withdrawal the subject from the study.
- If at any time a participant chooses to discontinue participation, they may do so with no repercussion to their care or future opportunities in research or other at the VA.

6.0 Reporting

- Because this is a low-risk trial of an educational DVD, we do not expect SAE's or AE's. We will plan to report any SAE's defined as death of participant or hospitalization, with data collected at 6-month follow-up. We will report SAEs identified in this manner within 5 business days of their identification. RA at site will notify the Local Site PI of any SAE and notify the Bedford project manager, who will then submit a Form 119 Unanticipated SAE to the appropriate SharePoint folder designated for Reportable AE/SAE.
- At the time of baseline visit, patients experiencing hypertensive urgency, as defined by JNC as Blood Pressure Systolic >= 180 OR Diastolic >= 120, regardless of symptoms, will be referred to urgent care and a notification letter will be sent to their primary care physician. All AE's reported at baseline and 6-month follow-up will be recorded by the RA and submitted to the Bedford Project manager, who will track and submit at Continuing Review.

7.0 Privacy and Confidentiality

- This study will obtain and keep confidential subjects' Protected Health Information (PHI).
- All study personnel with access to PHI will be required to maintain current required trainings and follow protocol for maintaining records according to best practices (i.e. locked file cabinet, locked safe when not on VA premises and Authorization to Transport on file, only study personnel access to drives or other e-locations of data)

8.0 Communication Plan

- The Coordinating site will be in charge of all CIRB communications and will assure submission of required documents. The Project manager will meet regularly with all sites to ensure local notification and R&D approvals are obtained and remain current.

VA Central IRB
Effective Date: June 6, 2022

- Any changes to the protocol or HIPAA documentation will be discussed with participating sites and submissions/approvals will be communicated to each site as they are completed an obtained.
- Any SAE or AE will be communicated from the Local Site and reported to the CIRB as outlined based on specifics of the events. Interim results and reports, such as DSMB will be forwarded to each of the participating sites in a timely manner.
- Each participating site will be provided with a copy of the approved protocol; and will receive updates at they occur.
- Bedford PI will communicate with the LSI's regarding timing of site closures.

9.0 References

REFERENCES

1. Whelton PK, Carey RM, Aronow WS, et al. 2017 ACC/AHA/AAPA/ABC/ACPM/AGS/APhA/ASH/ASPC/NMA/PCNA Guideline for the Prevention, Detection, Evaluation, and Management of High Blood Pressure in Adults: A Report of the American College of Cardiology/American Heart Association Task Force on Clinical Practice Guidelines. *J Am Coll Cardiol.* 2018;71(19):e127-e248.
2. Armstrong K, Putt M, Halbert CH, et al. Prior experiences of racial discrimination and racial differences in health care system distrust. *Medical care.* 2013;51(2):144-150.
3. Schoenthaler A, Montague E, Baier Manwell L, Brown R, Schwartz MD, Linzer M. Patient-physician racial/ethnic concordance and blood pressure control: the role of trust and medication adherence. *Ethn Health.* 2014;19(5):565-578.
4. Houston TK, Fix GM, Shimada SL, et al. African American Veterans Storytelling: A Multisite Randomized Trial to Improve Hypertension. *Medical care.* 2017;55 Suppl 9 Suppl 2:S50-S58.
5. Green MC, Brock TC. The role of transportation in the persuasiveness of public narratives. *J Pers Soc Psychol.* 2000;79(5):701-721.
6. Kreuter MW, Holmes K, Alcaraz K, et al. Comparing narrative and informational videos to increase mammography in low-income African American women. *Patient Educ Couns.* 2010;81 Suppl:S6-14.
7. Williams-Brown S, Baldwin DM, Bakos A. Storytelling as a method to teach African American women breast health information. *J Cancer Educ.* 2002;17(4):227-230.
8. Wolff M, Bates T, Beck B, Young S, Ahmed SM, Maurana C. Cancer prevention in underserved African American communities: barriers and effective strategies--a review of the literature. *WMJ.* 2003;102(5):36-40.
9. Kreuter MW, Holmes K, Hinyard LJ, et al. Narrative Communication in Cancer Prevention and Control: A Framework to Guide Research and Application. *Annals of Behavioral Medicine.* 2007;33(3):221-235.
10. Rubin AM. Parasocial Relationships. . *The International Encyclopedia of Interpersonal Communication*2015:1-5.

VA Central IRB
Effective Date: June 6, 2022
