# Supplementary material for: Text Messaging and Video Stories to Support Hypertension Self-Management in Black Veterans: A Randomized Clinical Trial
Source: JAMA Netw Open. 2025 Nov 5;8(11):e2541342. doi: 10.1001/jamanetworkopen.2025.41342 (PMC12590299; doi:10.1001/jamanetworkopen.2025.41342)
Supplement: Supplement 2. — eTable 1. Key Hypertension Self-Management Content Areas Incorporated Into Texting Protocols for Intervention and Control Group eTable 2. Example of the First 9 Weeks of Text Message Protocol for a Single Veteran Storyteller eTable 3. Demographic Characteristics: Comparison of Participants Completing Study to Those With Missing Follow-Up Data (Withdrawn or Lost to Follow-Up) eTable 4. Demographic Characteristics of Participants Missing Follow-Up Data (Withdrawn or Lost to Follow-Up) eTable 5. Supplemental Analysis Incorporating EHR-Derived Follow-Up Blood Pressure for Participants Missing or Lost to Follow-Up eTable 6. Descriptive Statistics and Difference-in-Differences Analysis for Additional Outcomes of ‘Continuing the Conversation’ (CTC) Randomized Controlled Trial eTable 7. Experiences and Reactions to Watching Video-taped Veteran Stories About Hypertension Self-Management [file jamanetwopen-e2541342-s002.pdf]

Supplemental Online Content

Cutrona SL, McDannold SE, DeLaughter KL, et al. Text messaging and video stories to support hypertension self-management in Black veterans: a randomized clinical trial. *JAMA Netw Open*. 2025;8(11):e2541342. doi:10.1001/jamanetworkopen.2025.41342

eTable 1. Key Hypertension Self-Management Content Areas Incorporated Into Texting Protocols for Intervention and Control Groups

eTable 2. Example of the First 9 Weeks of Text Message Protocol for a Single Veteran Storyteller

eTable 3. Demographic Characteristics: Comparison of Participants Completing Study to Those With Missing Follow-Up Data (Withdrawn or Lost to Follow-Up)

eTable 4. Demographic Characteristics of Participants Missing Follow-Up Data (Withdrawn or Lost to Follow-Up)

eTable 5. Supplemental Analysis Incorporating EHR-Derived Follow-Up Blood Pressure[a] for Participants Missing or Lost to Follow-Up

eTable 6. Descriptive Statistics and Difference-in-Differences Analysis for Additional Outcomes of 'Continuing the Conversation' (CTC) Randomized Controlled Trial

eTable 7. Experiences and Reactions to Watching Video-taped Veteran Stories About Hypertension Self-Management

This supplemental material has been provided by the authors to give readers additional information about their work.

© 2025 Cutrona SL et al. *JAMA Network Open*

eTable 1. Key hypertension self-management content areas incorporated into texting Protocols for Intervention and Control Groups[30]

Concept	Content Description
Salt Intake	Veteran describes strategies and contextually situated stories of how they managed salt intake
Talking with your doctor	Veteran describes reasons why honest communication with providers is important
Take your medicine	Veteran describes strategies and motivational tools in taking their prescribed medicine
Exercise	Veteran talks about specific strategies to increase exercise
Stress Management	Veteran describes tools and tactics to manage stress
Monitor your BP	Veteran describes ways they monitored their blood pressure
Diet/Nutrition	Veteran describes how they improved their diet and/or learned more about the importance of nutrition
Faith/Church/Community	Veteran shares how their faith, church or community are a source of support or motivation for them and their health
Alcohol/Smoking/Challenges	Veteran shares their experiences with alcohol, smoking, etc. and how they addressed these challenges

© 2025 Cutrona SL et al. *JAMA Network Open*

eTable 2. Example of the first 9 weeks of text message protocol for a single Veteran storyteller[30]

Title	Format	Day/ Time	Message*
Week 1: Salt Intake - Educational	Interactive (self-efficacy)	Day 1 11:00 AM	ANNIE-BP: How confident are you that you can make low salt choices shopping & eating? Text SAL 1 (not confident), SAL 2 (somewhat), or SAL 3 (very) to reply
	Educational	Day 2 3:00 PM	ANNIE-BP: To help control your blood pressure ('BP'), try to reduce the salt you eat. Diets high in salt can raise BP. Check nutrition labels for sodium (salt).
	Educational	Day 4 3:00 PM	ANNIE-BP: Processed foods, like frozen dinners, canned soup, and snacks, can have very high salt content. The DASH diet can help you make healthy choices.
	Educational	Day 6 3:00 PM	ANNIE-BP: You can also reduce salt by avoiding the saltshaker. Avoid sea salt & garlic salt too - try substitutes instead: Mrs. Dash or fresh herbs for flavor.
Week 2: Salt Intake - Narrative	Narrative	Day 8 3:00 PM	ANNIE-BP: Richard says: If you look at Campbell's soup, salt is one of its main ingredients. If you read labels, you can try to avoid foods with high salt
	Narrative	Day 10 3:00 PM	ANNIE-BP: Richard makes healthy choices to eat less salt. He says: I use Mrs. Dash's instead of salt. I do a lot of little stuff that adds up to big stuff.
	Narrative	Day 12 3:00 PM	ANNIE-BP: Richard noticed many foods have salt in them already: You can't get away from it so I decided not to add any more salt to my diet & I don't.
	Interactive (behavioral)	Day 13 1100	ANNIE-BP: How many DAYS this week did you make healthy salt choices in your diet? Text SALT 0, SALT 1, SALT 2 up to SALT 7 to reply.
Week 3: Exercise - Educational	Interactive (self-efficacy)	Day 15 11:00 AM	ANNIE-BP: How confident are you that you can make small daily changes to increase your activity? Text EX 1 (not at all), EX 2 (somewhat), or EX 3 (very) to reply.
	Educational	Day 16 3:00 PM	ANNIE-BP: Regular physical activity helps lower BP, & can help you reach or stay at a healthy weight. Talk to your care team about exercise that's right for you
	Educational	Day 18 3:00 PM	ANNIE-BP: Exercise doesn't have to be boring. You can ask a friend or co-worker to join you. Being active can help lower your BP and make you feel better too.
	Educational	Day 20 3:00 PM	ANNIE-BP: To increase your activity, you can walk, play a sport, do household chores, park further away from a store or take the stairs instead of the elevator.
Week 4: Exercise - Narrative	Narrative	Day 22 3:00 PM	ANNIE-BP: Exercise doesn't have to mean a big lifestyle change. Richard says, I do little stuff that adds up to big stuff and I do it every day.
	Narrative	Day 24 3:00:00 PM	ANNIE-BP: Find what works for you. Richard's knees made climbing stairs hard, so he said: Maybe I couldn't walk up 3 flights but I could definitely walk down 3.

© 2025 Cutrona SL et al. *JAMA Network Open*

	Narrative	Day 26<br>3:00 PM	ANNIE-BP: Find ways to add activity to your day. Richard said: Instead of parking right there I'm going to park over here in the back & walk to the front door.
	Interactive (behavioral)	Day 27<br>11:00 AM	ANNIE-BP How many DAYS this week have you done a specific exercise other than what you do around the house or at work? Text ACT 0, ACT 1, ACT 2 up to ACT 7 to reply
**Week 5: Take Meds - Educational**	Interactive (self-efficacy)	Day 29<br>11:00 AM	ANNIE-BP: How confident are you that you can take all your BP meds daily as prescribed? Text MED 1 (not at all), MED 2 (somewhat), or MED 3 (very) to reply.
	Educational	Day 30<br>3:00 PM	ANNIE-BP: Are BP meds part of your daily routine? If not, think of things you do regularly (toothbrushing, eating meals). Can these help you remember your meds?
	Educational	Day 32<br>3:00 PM	ANNIE-BP: Try reminder notes for meds - on the fridge, bathroom mirror, a pill box or a calendar (mark it after taking the meds), or set an alarm on your phone.
	Educational	Day 34<br>3:00 PM	ANNIE -BP: Plan ahead for refill requests so you don't run out. Talk to your VA care team if you have trouble getting your meds refilled on time.
**Week 6: Take Meds - Narrative**	Narrative	Day 36<br>3:00 PM	ANNIE-BP: Prescribed medication can be an important tool to help control your BP. This week we'll share Richard's tips to stay on track!
	Narrative	Day 38<br>3:00 PM	ANNIE-BP: Richard says, I take my med faithfully around the same time. You can't take it at 10 one day and then 2 the next. It doesn't get in your system right.
	Narrative	Day 40<br>3:00 PM	ANNIE-BP: Richard says: I take BP medicine once a day in the morning & what has happened is, the doctor's been able to reduce it because my BP has done so well.
	Interactive (behavioral)	Day 41<br>11:00 AM	ANNIE-BP: How many DAYS in the past week did you take your BP meds as prescribed? Text MEDS 0 through MEDS 7 to reply.
**Week 7: Faith/Church/ Community - Educational**	Interactive (self-efficacy)	Day 43<br>11:00 AM	ANNIE-BP: How confident are you that there are people to support you with your BP? Text SUP 1 (not at all), SUP 2 (somewhat), or SUP 3 (very) to reply.
	Educational	Day 44<br>3:00 PM	ANNIE-BP: Managing BP is hard. Reaching out to friends, family & community can help. This week think about where you can find support for important life changes
	Educational	Day 46<br>3:00 PM	ANNIE-BP: Sometimes, thinking of family & community can give you that spark of inspiration for making hard changes . Think about who inspires YOU to be healthy
	Educational	Day 48<br>3:00 PM	ANNIE-BP: The VA is part of your support system. Reach out to your healthcare team for support resources or visit https://www.va.gov/. We care!
**Week 8: Faith/Church/ Community - Narrative**	Narrative	Day 50<br>3:00 PM	ANNIE-BP: The Vets who shared their BP stories with us described talking to family or friends about their BP challenges. They asked for help staying on track.
	Narrative	Day 52	

© 2025 Cutrona SL et al. *JAMA Network Open*

		3:00 PM	ANNIE-BP: A Vet who shared his BP story with us says keeping healthy is a family effort: It's a long process. We're all trying to work together, help out.
	Narrative	Day 54<br>3:00 PM	ANNIE-BP: Some Veterans don't know they can get help at the VA – it can help you afford things like BP machines. Spread the word so VA can support other Veterans.
	Interactive (behavioral)	Day 55<br>11:00 AM	ANNIE-BP: In the past week have you gotten support from others as you work on healthy BP changes? Text SU-YES, SU-NO or SU-DIDN'T WANT to reply.
**Week 9: Check-In/ Motivational**	Interactive (self-efficacy)	Day 57<br>11:00 AM	ANNIE-BP: How confident are you in managing your BP? Text CONF 1 (not at all confident), CONF 2 (somewhat), or CONF 3 (very) to reply.
	Interactive (behavioral)	Day 59<br>11:00 AM	ANNIE-BP: How many DAYS this week did you make low salt choices when you bought food or when eating? Text SA 0, SA 1, SA 2, up to SA 7 to reply.
	Interactive (behavioral)	Day 60<br>11:00 AM	ANNIE-BP How many DAYS this week have you done a specific exercise other than what you do around the house or at work? Text MOV 0, MOV 1, MOV 2, etc to reply
	Interactive (behavioral)	Day 62<br>11:00 AM	ANNIE-BP: How many DAYS in the past week did you take your BP meds as prescribed? Text DAYS 0 through DAYS 7 to reply.

*Interactive text messages always include an acknowledgement of the response, usually with additional information on resources (not included in this table). For example, a response of 'SAL 3' to the Day 1 question on salt would receive the following response:

ANNIE-BP: That's great! If you'd still like to learn more about low salt choices, check out this info on the DASH diet. http://bit.ly/33Ru1XC

© 2025 Cutrona SL et al. *JAMA Network Open*

eTable 3. Demographic characteristics: Comparison of participants completing study to those with missing follow-up data (withdrawn or lost to follow-up)

	Missing follow-up (N=84)	Completed baseline and follow-up (N=516)	P value
**N=600**	**N (%)**	**N (%)**	
**Age**			0.45
30-49	2 (2.5)	29(5.7)	
50-69	55 (68.8)	325(64.2)	
70+	23 (28.8)	152(30.0)	
Total	80 (100.0)	506(100.0)	
**Sex**			0.31
Woman	14 (17.1)	113 (22.0)	
Male	68 (82.9)	401 (78.0)	
Total	82 (100.0)	514 (100.0)	
**Highest Education**			0.29
Some High School	2 (2.4)	15 (2.9)	
High School Graduate	29 (35.4)	131 (25.7)	
Some College	36 (43.9)	225 (44.1)	
Completed College	7 (8.5)	77 (15.1)	
Post Graduate Training	8 (9.8)	62 (12.2)	
Total	82 (100.0)	510 (100.0)	
**How Hard to pay for Basics**			0.00
Very hard	11 (13.8)	18 (3.6)	
Hard	12 (15.0)	37 (7.4)	
Somewhat hard	28 (35.0)	149 (29.7)	
Not very hard	29 (36.3)	298 (59.4)	
Total	80 (100.0)	502 (100.0)	
**How Hard to pay for Medical Care**			0.00
Very hard	11 (13.6)	18 (3.6)	
Hard	9 (11.1)	41 (8.2)	
Somewhat hard	16 (19.8)	71 (14.3)	
Not very hard	45 (55.6)	368 (73.9)	
Total	81 (100.0)	498 (100.0)	
**Combined Family Income**			0.61
$15,000 or less	11 (17.2)	64 (16.6)	
$15,001-20,000	9 (14.1)	43 (11.2)	
$20,001-$40,000	15 (23.4)	95 (24.7)	
$40,001-$60,000	17 (26.6)	75 (19.5)	
$60,001-$80,000	6 (9.4)	52 (13.5)	
$80,001 or more	6 (9.4)	56 (14.5)	
Total	64 (100.0)	385 (100.0)	

© 2025 Cutrona SL et al. *JAMA Network Open*

eTable 4. Demographic characteristics of participants missing follow-up data (withdrawn or lost to follow-up)

N=84	Intervention (N=38)<br>N (%)	Control (N=46)	P value
**Age**			0.86
30-49	1 (2.9)	1 (2.2)	
50-69	25 (71.4)	30 (66.7)	
70+	9 (25.7)	14 (31.1)	
Total	35 (100.0)	45 (100.0)	
**Sex**			0.44
Woman	5 (13.5)	9 (20.0)	
Male	32 (86.5)	36 (80.0)	
Total	37 (100.0)	45 (100.0)	
**Highest Education**			0.82
Some High School	1 (2.8)	1 (2.2)	
High School Graduate	13 (36.1)	16 (34.8)	
Some College	14 (38.9)	22 (47.8)	
Completed College	3 (8.3)	4 (8.7)	
Post Graduate Training	5 (13.9)	3 (6.5)	
Total	36 (100.0)	46 (100.0)	
**How Hard to pay for Basics**			0.46
Very hard	6 (16.7)	5 (11.4)	
Hard	7 (19.4)	5 (11.4)	
Somewhat hard	13 (36.1)	15 (34.1)	
Not very hard	10 (27.8)	19 (43.2)	
Total	36 (100.0)	44 (100.0)	
**How Hard to pay for Medical Care**			0.67
Very hard	6 (17.1)	5 (10.9)	
Hard	4 (11.4)	5 (10.9)	
Somewhat hard	5 (14.3)	11 (23.9)	
Not very hard	20 (57.1)	25 (54.3)	
Total	35 (100.0)	46 (100.0)	
**Combined Family Income**			0.35
$15,000 or less	8 (26.7)	3 (8.8)	
$15,001-20,000	4 (13.3)	5 (14.7)	
$20,001-$40,000	7 (23.3)	8 (23.5)	
$40,001-$60,000	8 (26.7)	9 (26.5)	
$60,001-$80,000	2 (6.7)	4 (11.8)	
$80,001 or more	1 (3.3)	5 (14.7)	
Total	30 (100.0)	34 (100.0)	

© 2025 Cutrona SL et al. *JAMA Network Open*

eTable 5. Supplemental Analysis incorporating EHR-derived follow-up blood pressure[a] for participants missing or lost to follow-up (n=38)

**Measure**	**CTC**		**BTM-Alone**		**Adjusted[b] Difference-in-Differences CTC vs. BTM-Alone with Confidence Interval**
**N=554**	*Baseline Mean (SD)*	*Follow-up Mean (SD)*	*Baseline Mean (SD)*	*Follow-up Mean (SD)*	
Systolic blood pressure (mmHg)	135(17)	133(17)	135(18)	134(15)	-0.9 [-3.9, 2.1]
Diastolic blood pressure (mmHg)	82(11)	80(11)	80(12)	78(12)	0.6 [-1.6, 2.6]

a. For 84 participants missing or lost to follow-up, we pulled from the EHR available systolic and diastolic blood pressures in a time window corresponding to that used for follow-up phone calls (2 weeks pre to 3 months post index date). Index dates were calculated as 6-months post the day on which texts were supposed to start for each participant. Of the 84 participants, 38 had EHR-recorded BP records in this time period. We selected systolic blood pressure and diastolic blood pressure closest to the index date in this time window (if multiple blood pressuress were recorded on the same day, we averaged them) and incorporated these office-based blood pressure values alongside home blood pressure checks (n=554 total) into a supplementary analysis.

b. Adjusted for correlation within Veterans

© 2025 Cutrona SL et al. *JAMA Network Open*

eTable 6: Descriptive Statistics and Difference-in-Differences Analysis for Additional Outcomes of 'Continuing the Conversation' (CTC) Randomized Controlled Trial

**Measure (range)**<br><br>**N=516**<br>**Shading indicates values showing change pre/post intervention**	**Intervention** Mean (SD)		**Control** Mean (SD)		**Adjusted[a] Difference-in-Differences** Intervention vs. Control
	Pre	Post	Pre	Post	
Range: 1 (Always) to 5 (Never)<br>**Lower value indicates better self-management unless otherwise indicated**					
I ask my doctor questions about my blood pressure medications	3(1)	2(1)	3(1)	3(1)	-.3 [-.8,.1]
I make efforts to maintain or reach a healthy weight	2(1)	2(1)	2(1)	2(1)	.01 [-.5,.5]
I make efforts to be physically active	2(1)	2(1)	2(1)	2(1)	-.3 [-.8,.2]
Since being told that I have high blood pressure, I eat healthier foods	2(1)	2(1)	2(1)	2(1)	.1 [-.4,.6]
I give up foods I enjoy in order to manage my high blood pressure	2(1)	2(1)	3(1)	2(1)	-.1 [-.5,.4]
I have someone check my blood pressure	4(1)	4(1)	4(1)	4(1)	.2 [-.3,.7]
I take blood pressure readings at home daily	3(1)	2(1)	3(1)	2(1)	-.3 [-.8,.1]
I eat ready to eat foods such as canned soup, frozen meals or take-out[b]	3(1)	4(1)	3(1)	4(1)	.3 [-.1,.8]

a. Adjusted for correlation within Veterans.
b. Higher value indicates better self-management for this question only

© 2025 Cutrona SL et al. *JAMA Network Open*

Measure (range)<br><br>N=516	**Intervention** Mean (SD)		**Control** Mean (SD)		**Adjusted[a] Difference-in-Differences** Intervention vs. Control
Questions begin with: ***How Confident are you that…*** *Not at all confident (1), somewhat confident (2), very confident (3);* Response options scored 1-3; ***Higher* value indicates higher confidence in self-management abilities**					
… you can make low salt choices shopping & eating?	3(1)	3(0)	3(1)	3(0)	-.2 [-.8,.5]
… you can exercise other than what you do at home or work?	3(1)	2(1)	2(1)	2(1)	-.4 [-1.0,.2]
… you can take all your BP meds daily as prescribed?	3(0)	3(0)	3(0)	3(0)	.3 [-.7,1.4]
… there are people to support you with your BP?	3(1)	3(0)	3(1)	3(0)	.2 [-.6,.9]
… in managing your BP?	3(0)	3(0)	3(0)	3(0)	.5 [-.2,1.2]
… in choosing healthy foods when you buy food and when you eat?	3(1)	3(1)	2(1)	3(1)	-.03 [-.6,.5]
…you can talk to your doc about your BP & BP med questions?	3(0)	3(0)	3(0)	3(0)	-.04 [-1.0,.9]
…you can use strategies for relaxing and reducing your stress?	3(1)	3(1)	3(1)	3(1)	.1 [-.5,.7]
…in avoiding challenges to BP control, like alcohol or smoking?	3(1)	3(0)	3(1)	3(0)	-.6 [-1.3,.1]
… in monitoring your BP at home?	3(0)	3(0)	3(0)	3(0)	.1 [-.6,.8]
Question below scored from 1 to 10 where 1='not at all confident' and 10='totally confident' ***Higher* value indicates higher confidence in self-management abilities**					
Having high blood pressure often means doing different tasks and activities to manage your condition. How confident are you that you can do all the things necessary to manage your high blood pressure on a regular basis?	9(2)	9(2)	9(2)	9(2)	-.3 [-1.0,.5]

© 2025 Cutrona SL et al. *JAMA Network Open*

a. Adjusted for correlation within Veterans.

**Measure (range)**<br><br>**N=516**	**Intervention** Mean (SD)		**Control** Mean (SD)		**Adjusted[a] Difference-in-Differences** Intervention vs. Control
Range: 1 to 5 (Strongly Disagree to Strongly Agree)<br>Higher value indicates stronger agreement, implications vary					
Having to take high blood pressure medicine worries me.	3(1)	2(1)	3(1)	3(1)	-.4<br>[-.9,.1]
I sometimes worry about becoming too dependent on my high blood pressure medicines.	3(1)	2(1)	3(1)	3(1)	-.4<br>[-.9,.1]
I sometimes worry about the long term effects of my high blood pressure medicines.	3(1)	3(2)	3(1)	3(1)	.1<br>[-.4,.6]
My high blood pressure medicines disrupt my life.	2(1)	2(1)	2(1)	2(1)	-.2<br>[-.7,.3]
My life would be impossible without high blood pressure medicines.	3(1)	3(1)	3(1)	3(1)	-.1<br>[-.5,.4]
My health, at present, depends on my high blood pressure medicines.	4(1)	4(1)	4(1)	4(1)	.1<br>[-.4,.6]
Without high blood pressure medicines, I would be very ill.	3(1)	4(1)	3(1)	4(1)	-.01<br>[-.5,.5]
My health, in the future, will depend on high blood pressure medicines.	4(1)	4(1)	3(1)	4(1)	-.1<br>[-.6,.3]
My high blood pressure medicines protect me from becoming worse.	4(1)	4(1)	4(1)	4(1)	.2<br>[-.3,.7]
My high blood pressure medicines are a mystery to me.	2(1)	2(1)	3(1)	2(1)	-.002<br>[-.5,.5]
If doctors had more time they would prescribe fewer high blood pressure medicines.	3(1)	3(1)	3(1)	3(1)	.3<br>[-.1,.8]

© 2025 Cutrona SL et al. *JAMA Network Open*

Doctors place too much trust in high blood pressure medicines.	3(1)	3(1)	3(1)	3(1)	.03 [-.5,.5]
Doctors use too many high blood pressure medicines.	3(1)	3(1)	3(1)	3(1)	-.04 [-.5,.4]
Natural remedies are safer than high blood pressure medicines.	3(1)	3(1)	3(1)	3(1)	.3 [-.2,.8]
Most high blood pressure medicines are addictive.	2(1)	2(1)	2(1)	2(1)	-.3 [-.8,.3]
High blood pressure medicines do more harm than good.	2(1)	2(1)	2(1)	2(1)	-.4 [-.9,.1]
All high blood pressure medicines are poisons.	2(1)	2(1)	2(1)	2(1)	-.1 [-.6,.4]
People who take high blood pressure medicines should stop their treatment for a while every now and then.	2(1)	2(1)	2(1)	2(1)	-.2 [-.7,.3]

a. Adjusted for correlation within Veterans.

eTable 7. Experiences and Reactions to Watching Video-taped Veteran Stories about Hypertension Self-Management[a]

N (%)	Not at all					Very Much	
**While I was watching the video, activity going on in the room around me** 242(100.0%)	203 (83.9%)	8 (3.3%)	8 (3.3%)	7 (2.9%)	4 (1.7%)	2 (.8%)	10 (4.1%)
**I was mentally involved in the video while watching it.** 241(100.0%)	8 (3.3%)	3 (1.2%)	4 (1.7%)	15 (6.2%)	14 (5.8%)	28 (11.6%)	169 (70.1%)
**The video affected me emotionally.** 242(100.0%)	56 (23.1%)	26 (10.7%)	20 (8.3%)	32 (13.2%)	33 (13.6%)	23 (9.5%)	52 (21.5%)
**I found my mind wandering while watching the video.** 242(100.0%)	175 (72.3%)	21 (8.7%)	11 (4.6%)	11 (4.6%)	7 (2.9%)	9 (3.7%)	8 (3.3%)
**The events in the video are relevant to my everyday life.** 237(100.0%)	17 (7.2%)	3 (1.3%)	6 (2.5%)	17 (7.2%)	37 (15.6%)	37 (15.6%)	120 (50.6%)

**The Veteran in this video…**	**Strongly Disagree**	**Disagree**	**Neither**	**Agree**	**Strongly Agree**
**Thinks like me.** 240(100.0%)	7(2.9%)	17(7.1%)	25(10.4%)	128(53.3%)	63(26.3%)
**Comes from a background like mine.** 237(100.0%)	14(5.9%)	34(14.4%)	31(13.1%)	110(46.4%)	48(20.3%)
**Has an education like mine.** 227(100.0%)	18(7.9%)	44(19.4%)	63(27.8%)	79(34.8%)	23(10.1%)
**Has values like mine.** 235(100.0%)	3(1.3%)	11(4.7%)	28(11.9%)	133(56.6%)	60(25.5%)
**Is a lot like me** 234(100.0%)	8(3.4%)	26(11.1%)	38(16.2%)	120(51.3%)	42(18.0%)
**I could identify with the Veteran in this video.** 239(100.0%)	1(.4%)	6(2.5%)	11(4.6%)	136(56.9%)	85(35.6%)

a. Participants were invited to choose their preferred storyteller (out of 5 whose videos they viewed) and they were asked to think of that person and video for the subsequent questions. Preferred storyteller (in order shown to participants): n (%): Veteran #1 = 90(35.9%); Veteran #2 =29(11.6%); Veteran #3 = 34(13.6%); Veteran #4 =60(23.9%); Veteran #5 = 38(15.1%).

© 2025 Cutrona SL et al. *JAMA Network Open*
