# Supplementary material for: Text Messaging and Video Stories to Support Hypertension Self-Management in Black Veterans: A Randomized Clinical Trial
Source: JAMA Netw Open. 2025 Nov 5;8(11):e2541342. doi: 10.1001/jamanetworkopen.2025.41342 (PMC12590299; doi:10.1001/jamanetworkopen.2025.41342)
Supplement: Supplement 3. — Data Sharing Statement [file jamanetwopen-e2541342-s003.pdf]

Data Sharing Statement

Cutrona. Text Messaging and Video Stories to Support Hypertension Self-Management in Black Veterans. *JAMA Netw Open*. Published November 05, 2025.
doi:10.1001/jamanetworkopen.2025.41342

Data

Additional Information: Clinical Trials Registration: IIR 17-185 Continuing the Conversation: Using Narrative Communication to Support Hypertension Self-management in African American Veterans, NCT03970590
URL:https://register.clinicaltrials.gov/prs/app/action/SelectProtocol?sid=S0008YUF&selectaction=Edit&uid=U0007OR7&ts=5&cx=6x2pik
Data available: No

Additional Information

Explanation for why data not available: Individual-level data on veterans cannot be made available to the public, due to VA regulation.
